# Phenotyping and Identification of Molecular Markers Associated with Leaf Rust Resistance in the Wheat Germplasm from Kazakhstan, CIMMYT and ICARDA

**DOI:** 10.3390/plants12152786

**Published:** 2023-07-27

**Authors:** Angelina Malysheva, Alma Kokhmetova, Rakhym Urazaliev, Madina Kumarbayeva, Zhenis Keishilov, Makpal Nurzhuma, Ardak Bolatbekova, Assiya Kokhmetova

**Affiliations:** 1 Institute of Plant Biology and Biotechnology, Almaty 050040, Kazakhstan; madina_kumar90@mail.ru (M.K.); jeka-sayko@mail.ru (Z.K.); maki_87@mail.ru (M.N.); ardashka1984@mail.ru (A.B.); asia.k68@mail.ru (A.K.); 2 Kazakh Research Institute of Agriculture and Plant Growing, Almalybak 040909, Kazakhstan; rakhim.urazaliyev@mail.ru

**Keywords:** wheat, *Triticum aestivum*, leaf rust, *Puccinia triticina*, resistance genes, molecular marker

## Abstract

Leaf rust (LR) is the most widespread disease of common wheat worldwide. In order to evaluate leaf rust resistance, 70 uncharacterized wheat cultivars and promising lines with unknown leaf rust resistance genes (*Lr* genes) were exposed to Kazakhstani *Puccinia triticina* (*Pt*) races at the seedling stage. Field tests were performed to characterize leaf rust responses at the adult plant growth stage in the 2020–2021 and 2021–2022 cropping seasons. The wheat collection showed phenotypic diversity when tested with two virulent races of *Pt*. Thirteen wheat genotypes (18.6%) showed high resistance at both seedling and adult plant stages. In most cases, breeding material originating from international nurseries showed higher resistance to LR. Nine *Lr* genes, viz. *Lr9*, *Lr10*, *Lr19*, *Lr26*, *Lr28*, *Lr34*, *Lr37*, *Lr46*, and *Lr68,* either singly or in combination, were identified in 47 genotypes. Known *Lr* genes were not detected in the remaining 23 genotypes. The most commonly identified resistance genes were *Lr37* (17 cultivars), *Lr34* (16 cultivars), and *Lr46* (10 cultivars), while *Lr19*, *Lr68*, *Lr26*, and *Lr28* were the least frequent. Four *Lr* genes were identified in Keremet and Hisorok, followed by three *Lr* genes in Aliya, Rasad, Reke, Mataj, Egana and Almaly/Obri. The molecular screening revealed twenty-nine carriers of a single *Lr* gene, ten carriers of two genes, six carriers of three genes, and two carriers of four genes. Most of these accessions showed a high and moderate level of APR (Adult plant resistance) and may be utilized for the incorporation of *Lr* genes in well-adapted wheat cultivars. The most effective combination was *Lr37*, *Lr34*, and *Lr68*, the carriers of which were characterized by a low disease susceptibility index. The obtained results will facilitate breeding programs for wheat resistance in Kazakhstan.

## 1. Introduction

Wheat (*Triticum aestivum* L.) is one of the world’s key grain crops and contributes significantly to food security. With over 781.38 million tons of annual production, wheat has become one of the most prevalent and important crops on the planet [1,2]. Every year in Kazakhstan, about 12.8 million hectares are allocated for sown areas, and 16–17 million tons of soft wheat are produced (stat.gov.kz (accessed on 20 April 2023)) [3]. The most prevalent disease in wheat is leaf rust, which is brought on by the parasitic basidiomycete *Puccinia triticina* [4]. Due to their small size, rust spores can be widely dispersed over wide geographic areas by wind [5]. Epidemics of leaf rust have been observed in all wheat-growing areas, including North and South America, Africa, Europe, Asia, and Australia [6,7,8,9,10,11]. The development of *P. triticina* on crops causes serious disease, resulting in yield losses reaching over 50% [12]. In Kazakhstan, widespread leaf rust is observed with an approximate frequency of once every 4 years, which was associated with an increase in the sown area of winter wheat [13]. Currently, moderate disease development of *P. triticina* is observed on the territory of Southern Kazakhstan [14]. In addition to leaf rust [15,16,17], diseases such as tan spots [18,19,20,21], yellow rust [22,23,24,25], and stem rust [26,27] are also widespread.

Wheat resistance to the leaf rust pathogen is associated with the presence of appropriate resistance genes. Each *Lr* gene is effective against a specific *P. triticina* race carrying the corresponding *Avr* gene. This interaction is called “gene for gene” [28]. The high evolutionary potential allows *P. triticina* to overcome the resistance of varieties carrying one or more resistance genes (R) to the most common races of the fungus in the region [29]. As a result of random mutations in clonal lines, recombinations involving the *Avr* genes, somatic hybridization, and geographical migration, new, more virulent races appear [6,30]. In addition, leaf rust spores are very viable and can persist for about one year, which allows them to winter quietly on crops [31].

According to the Catalogue of Gene Symbols, more than 80 *Lr* genes are already known to be distributed on all 21 wheat chromosomes; most of them come from wild relatives (alien species) [32]. For example, the *Lr9* gene was translocated into wheat from *Aegilops umbellulata* [33], and *Lr28*—from *Aegilops speltoides* [34]. Seedling resistance genes provide plant protection at all stages, also known as all-stage resistance (ASR) [7]. ASR genes are expressed throughout the life of the plant [35]. This type of resistance is race-specific [36]. Two genes are needed to express resistance: the ASR gene in the host and the corresponding avirulence (Avr) effector gene in the rust pathogen. Each ASR gene confers resistance to pathogen strains carrying the corresponding Avr effector gene [37]. Examples of race-specific genes are *Lr1*, *Lr10*, and *Lr21*. These genes cause a low level of infection, manifested by the appearance of hypersensitivity patches or small uredinia encircled by chlorosis or necrosis [38]. The long-term effectiveness of seedling resistance genes depends on how widely varieties carrying these genes are cultivated [39].

Adult plant resistance (APR) genes are expressed at post-emergence stages [30]. The key characteristic of APR genes is that they confer resistance to all known races of *P. triticina.* The single APR genes are not able to completely prevent the formation of urediniums and provide an immune response [4]. APR genes cause long-term resistance and slow down the development of rust diseases. *Lr34*, which is present in wheat germplasm all over the world [40], is the most well-known and well-studied of these genes [41]. The *Lr34* gene encodes an ATP-binding cassette transporter protein (ABC transporters) and might be involved in the secretion of antifungal molecules. The proteins of this family have a similar basic structure, consisting of two cytosolic nucleotide-binding domains and two hydrophobic transmembrane domains. Identical homologous proteins were found in rice (OsPDR23) and Arabidopsis [42]. There are complicated loci in several APR genes that also confer resistance to stem rust (*Sr*), yellow rust (*Yr*), and powdery mildew (*Pm*)—*Lr34*/*Sr57*/*Yr18*/*Pm38* [43], *Lr37*/*Yr17*/*Sr38* [44], and *Lr67*/*Sr55*/*Yr46*/*Pm46* [45]. The use of APR genes along with four to five ASR genes is a cost-effective and environmentally friendly wheat protection strategy that provides long-term resistance [46].

Gene pyramiding can be carried out both by traditional breeding methods and by indirect selection using DNA markers associated with resistance genes [47]. Traditional breeding is highly dependent on environmental conditions and time constraints. So, the breeding of a new variety takes from 8 to 12 years. Molecular markers overcome these limitations and are used to identify and map resistance genes on wheat chromosomes [48,49]. Molecular markers are actively and effectively used in the breeding programs of many developed countries. Wheat varieties developed at CIMMYT with combinations of adult plant genes *Lr34*, *Lr46*, and *Lr68* have shown long-term resistance. It was also shown that it is still effective to use varieties and lines with the genes *Lr9*, *Lr10*, *Lr19*, *Lr26*, *Lr28*, *Lr34*, *Lr37*, and *Lr68* to control the Kazakhstan leaf rust populations [17,50]. The present study was conducted to determine leaf rust responses and identify sources of effective *Lr* genes in the diverse wheat germplasm from Kazakhstan as well as advanced lines originating from the breeding program IWWIP (International Winter Wheat Improvement Program) developed by CIMMYT-ICARDA.

The purpose of this study was to assess a collection of winter wheat for LR seedling and adult plant resistance (APR) and to investigate the potential for resistance in wheat germplasm using molecular markers linked to *Lr* genes.

## 2. Results

### 2.1. Reaction of the Wheat Collection to Two Races of P. triticina at the Seedling Stage

The ANOVA results revealed highly significant variation (*p* < 0.001) for wheat genotypes, while the race effect was found to be significant (*p* < 0.001) (Table 1).

**Figure 1:**
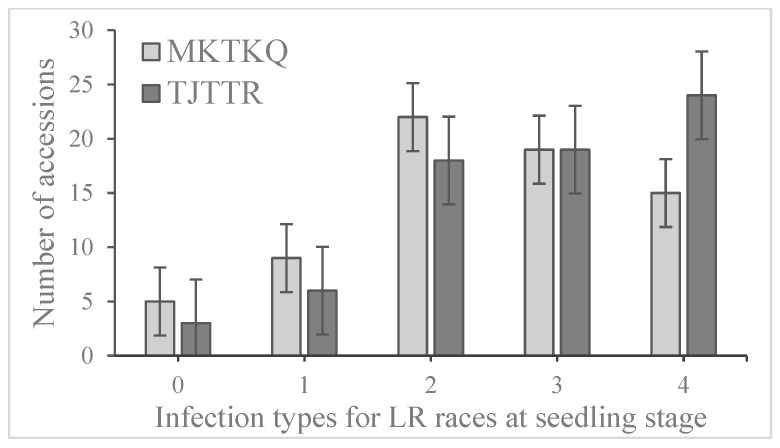
Frequency of 70 winter wheat genotypes in different disease score groups when tested with *Puccinia triticina* races TJTTR and MKTKQ. Note: 0—immune, 1—resistant, 2—moderately resistant, 3—moderately susceptible, and 4—susceptible.

A wide variation was observed in leaf rust disease severity based on infection types (IT) 0–4, ranging from very resistant to very susceptible in the wheat collection. Further, the frequency distribution of the infection types (ITs) produced by the two different *Pt* races (TJTTR and MKTTQ) for resistant and susceptible genotypes based on mean values is shown in Figure 1. Among 70 wheat accessions, 43 (61.4%) showed susceptibility to the TJTTR race (IT = 3–4). Nine cultivars (16.4%) showed high seedling resistance to TJTTR. Among them, three cultivars (Azharly, Keremet and Shafag 2) showed an immune reaction (IT = 0), and six cultivars (Bunyodkor, Hisorok, Layagatlii 80, Egana, Naz/GF552 and 7-CP) showed a resistance reaction (IT = 1).

Thirty-six accessions (51.4%) demonstrated resistant or moderately resistant reactions to *Pt* race MKTKQ (IT—0–2). Five cultivars (Azharly, Keremet, Shafag 2, 7-CP and 13-CP) showed immune (IT = 0) reactions, and nine cultivars (Rasad, Alihan, Almaly/Obri, Bunyodkor, Hisorok, Layagatlii 80, 416-SP-2, 6-CP and 9-CP) showed resistance (IT = 1) reactions (Table 2). A higher percentage of wheat accessions were susceptible (IT = 3–4) to the race TJTTR (61.4%) as compared to the race MKTKQ (48.6%). Among the genotypes, a high level of heritability (h_b_^2^) for leaf rust resistance was revealed (0.90).

Reactions of wheat seedlings to race TJTTR were strongly associated with resistance to race MKTKQ (r = 0.86; *p* < 0.001). Correlation analysis between the response of seedlings to race TJTTR and the average coefficient of infection (ACI) showed a significant positive correlation in 2021 (r = 0.44; *p* < 0.001) and 2022 (r = 0.58; *p* < 0.001). This analysis revealed a positive relationship between the leaf rust average coefficient of infection (ACI) and responses to the race MKTKQ in 2021 (r = 0.51; *p* < 0.001) and in 2022 (r = 0.64; *p* < 0.001).

### 2.2. Field Evaluation

**Figure 2:**
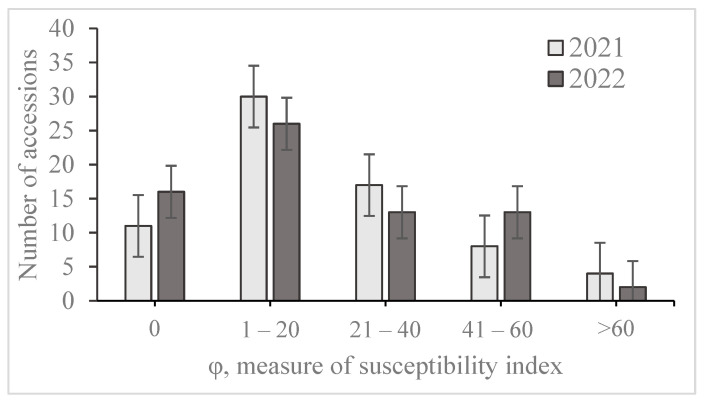
Distribution of the winter wheat collection according to the values of the index of susceptibility to leaf rust in the field. Note: Susceptibility is on the horizontal axis and is a measure of the susceptibility index (φ), calculated from the ratio of the AUDPC of the accession to the AUDPC of the susceptible control. The number of accessions is on the vertical axis.

A total of 70 wheat genotypes were evaluated for leaf rust resistance in field tests and ranked into a group of resistant (0, R-MR) and susceptible (MS-S) accessions (Table 2). The disease severity in 2021 ranged from 0 (Keremet, Gozgon, Rasad, Hisorok, Egana, Alihan, 416-SP-2, 7-CP, 12-CP, 13-CP and 17-CP) to 96% (Daulet). The cultivars Keremet, Karlygash, Egemen 20, Rasad, Naz/GF55-2, Hisorok, Layagatlii 80, Egana, Almaly/Obri, Gozgon, Alihan, 416-SP-2, 7-CP, 9-CP, 10-CP and 13-CP was recorded as immune genotypes with disease severity of 0% in 2022. The maximum disease severity observed for the cultivar Aliya was 83%. A histogram for the number of accessions scored at each value is shown in Figure 2. The distribution of mean leaf rust susceptibility frequencies was continuous, indicating quantitative inheritance. In 2021, leaf rust severely affected wheat cultivars Rausin, Daulet, Derbes, and Naz/Immun 78 with the highest AUDPC, while in 2022, the cultivars Aliya and Naz/Immun 78 had the highest AUDPC (Table 2). The susceptibility index made it possible to group the wheat genotypes according to their severity. The group with a severity index of 1–20% prevailed in both years (Figure 2). Leaf rust severity was significantly different across genotypes in both growing seasons, according to an ANOVA (*p* < 0.001) analysis. The high level of heritability (h_b_^2^—0.79) of the disease susceptibility index among wheat genotypes was shown (Table S1).

To identify the most promising wheat varieties, an analysis of productivity was carried out, which made it possible to evaluate the wheat collection based on the plant height (PH, cm), the days to heading (DH), the spike lengths (SL, cm), the mean number of spikelets/spike (SS), the number of grains per spike (GS), the weight of grain per spike (WGS, g), and the thousand kernel weight (TKW, g) (Table S2). The largest difference of 19 days was observed between the wheat cultivars Matai (228 days) and 14-CP (209 days) for DH in 2021, and 14 days between Almaly (232 days) and 4-CP (218 days). The mean PH ranged from 73 to 137 cm in 2021 (Hisorok was the shortest; 9-CP and 3-CP were the tallest); and from 60 to 125 cm in 2022 (Konditerskaya and Naz/GF55-2, respectively). The mean TKW ranged from 27.9 (Daulet) to 52.6 g (Koksu) in 2021 and from 29.4 (Farabi) to 48.6 g (Egemen) in 2022. The most productive accessions include Kyzyl Bidaj, Progress, Akbidaj, 428/MK-122A-1, Gozgon, Alihan, 4-CP, 6-CP, 3-CP, 7-CP, Alatau, Koksu in 2021, and 9-CP, Kazakhstanskaya 10, Almaly/Obri, Kokbidaj, Rasad, Koksu, Alihan, Gozgon and Egemen 20 in 2022. Statistical analysis revealed significant differences among genotypes for most of the analyzed agronomic traits for both growing seasons (Table S1). A high level of heritability was noted for plant height (0.66), spike length (0.75), and thousand kernel weight (0.74).

There was a significant negative correlation in 2021 between AUDPC and TKW (r = −0.6; *p* < 0.001), as well as between AUDPC and WGS (r = −0.48; *p* < 0.001) and AUDPC and GS (r = −0.40; *p* < 0.001). Analysis between NDVI and DH has shown significant positive correlations (r = 0.25; *p* < 0.05), as well as between NDVI and PH (r = 0.38; *p* < 0.05) (Figure S1). In 2022, AUDPC was negatively correlated with WGS (r = −0.44; *p* < 0.001), GS (r = −0.27; *p* < 0.01), and TKW (r = −0.55; *p* < 0.001). A positive correlation was noted between NDVI and DH (r = 0.19; *p* < 0.05).

**Figure 3:**
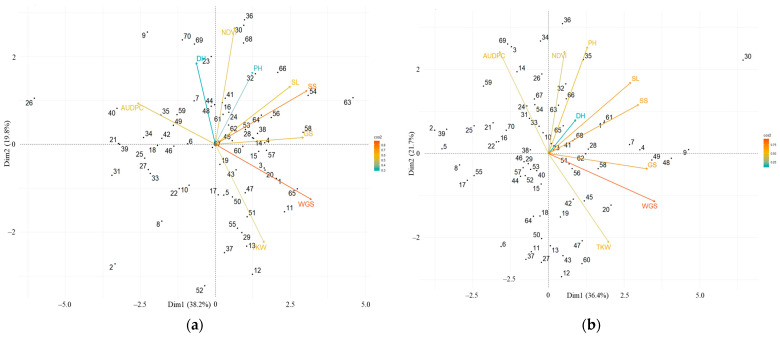
Principal component analysis biplots for 70 winter wheat accessions based on leaf rust severity and productivity traits in 2021 (**a**) and 2022 (**b**). Note: PH, plant height; DH, the days to heading; SL, the spike lengths; SS, the mean number of spikelets/spike; GS, the number of grains per spike; WGS, the weight of grain per spike; TKW, the thousand kernel weight; AUDPC, area under the disease progress curve; NDVI, normalized difference vegetation index.

In order to examine the association between traits, a principal component analysis (PCA) was performed and visualized as separate biplots for 2021 and 2022 (Figure 3). PCA was performed based on the results of the AUDPC parameters and yield components. This analysis showed that the first two principal components explained 58% of the variation in 2021. The first principal component accounted for 38.2% of the variations. The WGS, SS, GS, AUDPC, and SL parameters made the greatest contribution to PC1. All spike productivity traits were closely correlated. The second principal component (PC2) explained 19.8% of the variation and combined the effects of NDVI, TKW, DH, and PH. In 2022, the first two main components explained 58.1% of the variation. PC1 (36.4%) combined the effects of WGS, GS, SS, and SL. The greatest contribution to PC2 (21.7% variation) was made by traits PH, NDVI, AUDPC, and TKW. All traits of productivity were closely correlated. AUDPC had a significant negative effect on TKW and WGS in both growing seasons.

Biplot analysis, based on the reaction of wheat accessions to the leaf rust pathogen and productivity traits, showed that the samples Rasad, Hisorok, Koksu, Gozgon, Kokbidaj, and Alihan showed the most resistant reaction to the pathogen and high productivity.

### 2.3. Identification of Lr Resistance Genes Using Molecular Markers

In order to test 70 varieties and lines of winter wheat, nine closely linked specific markers for nine investigated *Lr* genes were individually identified in each corresponding NIL (*Lr9*, *Lr10*, *Lr19*, *Lr26*, *Lr28*, *Lr34* and *Lr37*), as well as in cv Pavon 76 (*Lr46*) and in cv. Parula (*Lr68*). Molecular screening results for the presence of relevant *Lr* genes are presented in Table 2 and Figure S2–S10. The STS J13 marker was used, amplifying the 1100-bp product to search for carriers of the *Lr9* gene. The marker is closely linked to the gene, as evidenced by the absence of recombination between them [51]. Of the 55 tested samples in the collection, the expected marker fragment associated with *Lr9* was found in seven of the fifty-five genotypes, including Mataj, Raminal, Yzhnaya 12, Hisorok, Egana, Anar, and Almaly/Obri.

The search for the *Lr10* gene was carried out using the specific STS marker FI.2245lr10-6/r2 [52]. The marker fragment specific to *Lr10* was found in six wheat accessions: Aliya, Hazarti Bashir, Hisorok, Layagatlii 80, Egana, and Yr2/Octyabrina.

Common wheat received a translocation from *Agropiron elongatum* (Host) Beauvois with the gene *Lr19* located on chromosome 7DL. This translocation is also associated with the yellow coloration of the endosperm, which limits its use in bread wheat breeding. Zhang and Dubcovsky developed a set of markers for alleles of the phytoene synthase 1-Psy-B1 gene, which also allows the detection of the presence of *Lr19* [53]. The marker fragment linked to the *Lr19*/*Sr25* gene complex was detected in three wheat accessions (Kyzyl bidaj, Keremet, and Hisorok), as evidenced by the presence of a 191 bp PCR product.

The genes for resistance to leaf (*Lr26*), stem (*Sr31*), and yellow (*Yr9*) rust are located on the short arm of chromosome 1 of rye (1RS) and have been transferred to wheat through translocations [54]. The STS marker Iag95 was mapped as a codominant marker 1.99 cM distal to the leaf rust resistance gene *Lr26* [55]. The presence of the *Lr26* gene was confirmed by amplification of the 1100 bp product in eight genotypes: Keremet, Bunyodkor, Shafag 2, Layagatlii 80, Naz/GF55-3, 416-SP-2, 9-CP and 14-CP.

The SSR marker WMC 313 linked to *Lr28* at a distance of 5.0 cM was used to identify this gene [56]. The *Lr28* gene was transferred to wheat from *Aegilops speltoides Tausch* and is located on the long arm of chromosome 4A [57]. A 320-bp amplification product indicating the presence of *Lr28* was detected in five wheat accessions (Aliya, Reke, Pirotrix 50, Konditerskaya, and 425/Obri).

The *Lr34* gene is linked to the R genes for yellow rust, *Yr18*; stem rust, *Sr57*; and powdery mildew, *Pm38*. The presence of the APR gene *Lr34*/*Yr18* was assessed using the codominant STS marker csLV34, linked at a distance of 0.4 cm from the *Lr34* locus [43]. The 150 bp amplification product indicated the presence of *Lr34* and was found in 16 wheat accessions, including Almaly, Karaspan, Karlygash, Raminal, Rasad, Egana, Naz/GF55-2, Almaly/Obri, 416-SP-2, 2-CP, 4-CP, 5-CP, 15-CP, 16-CP, 17-CP, and 19-CP.

The *Lr37*/*Sr38*/*Yr17* gene complex localized in the short arm of the 2NS chromosome of *Triticum ventricosum* (*Tausch*) was translocated to the short arm of the common wheat chromosome 2AS. The CAPS marker Uric-Ln2 was used to identify wheat genotypes carrying the 2NS translocation [44]. The *Lr37* gene was found in 17 wheat cultivars, including Aliya, Matai, Kyzylbidai, Keremet, Kokbidai, Rasad, Reke, Hisorok, Alikhan, Krasnovodopadskaya 210, 3-CP, 7-CP, 9-CP, 11-CP, 14-CP, 15-CP, and 17-CP.

The *Lr46* gene, which is a complex locus providing multifactorial resistance to yellow rust (*Yr29*), stem rust (*Sr58*), and powdery mildew (*Pm39*) [58], had a high frequency of occurrence (20%). Identification of the sources of this gene complex was performed using the STS marker Wmc44. This microsatellite locus was mapped 5–15 cM proximal to *Lr46* [59]. Screening of wheat collections revealed 10 carriers of *Lr46*, including cvs Alatau, Azharly, Akbidaj, Batyr, Farabi, Mataj, Keremet, Koksu, Rasad, and Reke.

The marker linked to *Lr68* was found in three wheat genotypes: Naz/GF55-3, Alihan, and Almaly/Obri. Carriers of the resistance gene were identified using the dominant STS marker csGS, mapped at a distance of 1.2 cM proximal to *Lr68* [60].

## 3. Discussion

Rust diseases were and still are one of the key reasons for the decline in yields and deterioration in the quality of wheat grain both in Kazakhstan [13] and around the world [61]. The incidence of leaf rust in Central Asia is associated with sources of infection, weather conditions, and cultivar resistance [62]. The leaf rust population in Kazakhstan has a wide range of virulence, varies by region, and is subject to change [17]. The isolates collected from the affected plants of Northern Kazakhstan are similar in virulence to the population of Western Siberia [63]. Extensive studies of the population structure revealed that all pathotypes isolated in the regions of Western Siberia, the Urals, and Northern Kazakhstan were avirulent to the *Lr19* and *Lr24* genes [64,65]. Earlier, it was also reported about the avirulence of the Kazakhstan South-Eastern population to the genes *Lr9*, *Lr19*, *Lr24*, *Lr25*, and *Lr28*, and that of the North Kazakhstani population to *Lr19*, *Lr24*, *Lr25*, *Lr28*, *Lr36* and *Lr45* [50]. In our study, avirulence to *Lr9* and *Lr19* was confirmed for both races of the pathogen on Thatcher differential lines, which indicates the effectiveness of these genes in providing seedling resistance.

The resistance of 70 wheat genotypes to the pathogen *P. triticina* that causes leaf rust was assessed in this study during the seedling and adult plant stages. One of the primary objectives of breeding programs is the identification of resistant genotypes [66,67]. According to the reaction to the leaf rust pathogen, the studied wheat collection showed genotypic diversity. Thirty-six of the studied genotypes showed a stable response to the MKTKQ race, and twenty-seven genotypes were resistant to the TJTTR. In 26 wheat accessions, simultaneous resistance to both races was found. Thirteen varieties of them showed the resistance of adult plants (Keremet, Rasad, Naz/GF55-2, Alihan, Gozgon, Hisorok, Egana, 415-SP-2, 416-SP-2, 7-CP, 10-CP, 13-CP, and 17-CP). The majority of wheat genotypes (18.6%) showed high resistance at both seedling and adult plant stages. In most cases, breeding material originating from international nurseries (IWWIP, KZ-CIMMYT) showed higher resistance to LR. Eleven cultivars (Alatau, Batyr, Kokbidaj, Faravon, Anar, 2-CP, 3-CP, 5-CP, 11-CP, 12-CP, and 15-CP) were sensitive at the seedling stage but showed adult plant resistance (ϕ—0–20) and can be considered sources of APR genes.

Molecular screening of seventy wheat accessions was conducted with linked nine markers to identify both carriers of single resistance *Lr* genes and gene complexes. A total of 47 carriers of resistance genes were identified. In varieties Egemen 20, Kazakhstanskaya 10, Progress, Prezident, Rausin, Zhalyn, Daulet, 428/MK-122A-1, Faravon, Steklovidnaya 24, Sultan 2, Naz/Immun 78, Naz/GF55-5, Derbes, APK/Progress, Gozgon, 415-SP-2, 6-CP, 8-CP, 10-CP, 12-CP, 13-CP, and 18-CP, none of the tested *Lr* genes were identified. The Gozgon (5R), 13-CP (IT-0), 17-CP (20MR), and 415-SP-2 (10MR) genotypes demonstrated a high level of resistance to *P. triticina*, indicating that extra *Lr* genes are giving resistance in these genotypes.

The origins of *Lr* resistance genes in wheat breeding material were discovered in several earlier studies [16,17,50,68]. The genetic screening of spring wheat cultivars for this study revealed variations in the frequencies of nine crucial *Lr* genes. Twenty-nine cultivars with one *Lr* gene were identified. Ten accessions of wheat were carriers of two *Lr* genes. Among the 70 accessions produced in Kazakhstan, three leaf rust resistance genes (*Lr37*, *Lr34* and *Lr46*) were demonstrated to occur at high frequency: 24.3%, 22.8% and 14.3%, respectively.

Seven (10%) carried the leaf rust resistance gene *Lr9*, six (8.6%) carried the gene *Lr10*, and three accessions (4.3%) had *Lr19* and *Lr68* each; *Lr26* and *Lr28* were found in eight (11.4%) and five (7.1%) cultivars, respectively. These leaf rust resistance genes showed evidence of providing adequate protection in the investigated genotypes. Two genes (*Lr9* and *Lr34*) were identified in Raminal; *Lr10* and *Lr26* were found in Layagatlii 80; *Lr37* and *Lr19*—in Kyzylbidaj; *Lr26* and *Lr68*—in Naz/GF55-3; *Lr68* and *Lr37*—in Alihan; *Lr26* and *Lr34*—in 416-SP-2; *Lr26* and *Lr37* in 9-CP and 14-CP; *Lr34* and *Lr37*—in 15-CP and 17-CP.

The *Lr37* and *Lr34* genes differed in the highest frequency of occurrence (24.3 and 22.8%, respectively). The *Lr37* gene still provides a sufficient level of resistance, which indicates the need for its introduction into breeding programs [69,70,71,72,73]. The *Lr46* gene provides “slow-rusting”, although it is a less effective gene compared to the *Lr34* gene. When combined with *Lr34* and/or *Lr68*, it provides an almost immune response to the leaf rust pathogen [74]. Race non-specific resistance is more effective at the stage of an adult plant. This is due to a longer latency period, a low infection rate, a shorter duration of sporulation, and less sporulation [75]. *Lr34* is the first cloned slow-rusting gene that has been stable for over 50 years [76]. The effectiveness of the *Lr19* gene against leaf rust races and the *Sr25* gene against stem rust was previously confirmed in an extensive collection of wheat germplasm [77]. *Lr9*, *Lr19*, and *Lr28* genes are still effective in China [78], India, Nepal, and Bangladesh [79], Slovakia [80], Iran [81], France [82], Egypt [66,83], and Northwest Russia [84], but pathotypes were identified that overcame resistance to *Lr9* and *Lr19* in Bulgaria [85], and *Lr28* in the US of America [86]. Also, resistance to *Lr26* was overcome in Chinese and Indian leaf rust populations [78,79].

In our study, the most effective combination was the presence of *Lr37*, *Lr34*, and *Lr68*, the carriers of which were characterized by a low disease susceptibility index (φ—0). In six varieties, three resistance genes were found: *Lr10*, *Lr28*, *Lr37*—in Aliya; *Lr34*, *Lr37*, *Lr46*—in Rasad; *Lr28*, *Lr37*, *Lr46*—in Reke; *Lr9*, *Lr37*, *Lr46*—in Mataj; *Lr9*, *Lr10*, *Lr34*—in Egana; *Lr9*, *Lr34*, *Lr68*—in Almaly/Obri. Two cultivars were carriers of four resistance genes: *Lr37*, *Lr26*, *Lr46* and *Lr19* (Keremet); *Lr9*, *Lr10*, *Lr37* and *Lr19* (Hisorok). These cultivars showed a high level of resistance at the stage of an adult plant for the entire growing season (φ—0). Different responses of cultivars carrying the same resistance genes to *P. triticina* may be associated with the cumulative effect of genes, the presence of unidentified genes, different expression levels of resistance genes, and other biotic and abiotic factors [87,88]. Future breeding initiatives can take advantage of the findings of this investigation; sources of race-specific and non-race-specific genes can be used for pyramiding with other effective *Lr* genes.

## 4. Materials and Methods

### 4.1. Plant Material

The collection of 70 winter wheat genotypes (*Triticum aestivum* L.), including 42 cultivars grown and/or produced in Kazakhstan, 8 cultivars/advanced lines originating from the breeding program IWWIP (International Winter Wheat Improvement Program) developed by CIMMYT-ICARDA, and 20 advanced lines selected from the Kazakhstan-CIMMYT breeding program (Table S3), was used in this study. The highly susceptible cultivar Morocco was used as the negative controls; resistant check Pavon 76 carrying *Lr46* and resistant check Parula carrying *Lr68*, as well as the *Lr* gene near-isogenic lines (NIL), Transfer/6*TC (*Lr9*), TC*6/Exchange (*Lr10*), TC*7/Tr (*Lr19*), TC*6/ST-1-25 (*Lr26*), CS2D-2M (*Lr28*), TC*6/PI58548 (*Lr34*), and TC*6/VPM (*Lr37*) were used as positive controls in *Lr* gene detection.

### 4.2. Leaf Rust Spore Collection, Multiplication and Race Identification

Spore collection, storage and reproduction were performed under controlled conditions at the Institute of Plant Biology and Biotechnology, Almaty, Kazakhstan. Leaves bearing the uredinia of leaf rust, *Puccinia triticina,* were collected in 2020 from common wheat, including the experimental plots and commercial fields in the Almaty region. Three to ten leaves of a single variety from each plot/field were considered one sample. Infected leaves were air-dried and stored at 4 °C until spores were collected for inoculation and increase. Up to two single uredinial isolates were derived from each rust sample and tested for infection type. Leaf rust uredinia from dry leaves were renewed on a susceptible cultivar in Morocco, and single pustule isolates were obtained. Multiplication of single urediniospore isolates for virulence tests was performed using detached leaf segments preserved in a water–benzimidazole solution (40 mg/L) [89]. Leaf segments were incubated at 100% relative humidity and 19–22 °C in darkness for 18 h, followed by 20 °C with 18 h of light. Using a spray gun, leaves of cultivar Morocco were inoculated with urediniospores suspended in light mineral oil at a concentration of 2–3 mg/mL (5–10 × 10^3^ spores). Pustules of leaf rust appeared on the leaves 8–10 days after inoculation, from which inoculum was collected on the 12th day using a mechanical cyclone collector in a zero-size capsule. Spores were dried up to 20–30% relative humidity and then sealed in glass vials. The spores were preserved in an ultra-low refrigerator at −70 °C until further use. Urediniospores were heat treated in a water bath at 40 °C for 5–7 min to break cold-induced dormancy upon removal from storage [90,91].

Races of *P. triticina* were differentiated using the three-letter nomenclature of Long and Kolmer (1989), which served as the basis for the virulence codes for the isolates [92]. Virulence phenotypes were determined on the set of 20 near-isogenic lines (NIL) cv. Thatcher [93]. Set 1: *Lr1* (RL6003), *Lr2a* (RL6000), *Lr2c* (RL6047), and *Lr3* (RL6002); set 2: *Lr9* (RL6010), *Lr16* (RL6005), *Lr24* (RL 6064), and *Lr26* (6078); set 3: *Lr3ka* (RL6007), *Lr11* (RL6053), *Lr17* (RL6008), and *Lr30* (RL6049); set 4: *Lr2b* (RL6019), *Lr3bg* (RL6042), *Lr14a* (RL6013), and *Lr14b* (RL6006); set 5: *Lr15* (RL60052), *Lr18* (RL6009), *Lr19* (RL6040), and *Lr20* (RL6092). For all seedling tests, seeds were sown in 12-cm pots and placed at 18 °C until germination. Using a spray gun, seedlings at the two-leaf stage were inoculated with urediniospores from individual rust samples suspended in light mineral oil at a concentration of 2–3 mg/mL (5–10 × 10^3^ spores). The inoculated seedlings were kept for a day in a climatic chamber at 70% humidity and a temperature of 18°, followed by 20 °C with 18 h of light [93]. The seedling resistance of the NIL collection to the isolates of leaf rust was assessed 10–12 days after inoculation according to the Mains and Jackson scale [94]. Infection types (IT) 0–2+ (immune response to moderate uredinia with necrosis and/or chlorosis) were classified as avirulent, and infection types (IT) 3–4 (moderate to large uredinia without chlorosis or necrosis) were classified as virulent. Reaction types of 20 differentials were encoded and designated by a letter using the code according to the corresponding binary quadruple. Then each isolate was given a five-letter code (one letter for each set of four differentials), as adapted from the North American nomenclature for virulence in *Puccinia triticina* [93]. Cultivar Thatcher was included in experiments as a susceptible control. The virulence/avirulence analysis of pathogen isolates is presented in Table 3.

The TJTTR race was characterized by high virulence (85%) and was more aggressive than the MKTKQ race (70%).

### 4.3. Leaf Rust Evaluation at the Seedling Stage

A collection of 70 varieties and lines of winter wheat was screened at the seedling stage for two leaf rust races. Plant reactions to leaf rust at the seedling stage were evaluated for the two *P. triticina* isolates, TJTTR and MKTKQ. Under laboratory conditions, the plants were grown in plastic containers (5–8 grains of each cultivar). At the first leaf phase (10–12 days), plants were sprayed with each race’s spore suspension at a concentration of 2–3 mg/mL (5–10 × 10^3^ spores). The incubation of infected plants was carried out according to the parameters described above for race identification. The seedling resistance of wheat collected to the isolates of leaf rust was assessed 10–12 days after inoculation according to the Mains and Jackson scale [94].

### 4.4. Leaf Rust Evaluation at the Adult Plant Stage

The experimental material was phenotyped during the 2021 and 2022 growing seasons at the Kazakh Research Institute of Agriculture and Plant Growing (KRIAPG), Almalybak (43°13′ N, 76°36′ E, and 789 masl), Almaty region. Three replicates were used in a totally randomized design for the experiment. The individual plot size was 1 m^2^. Treatments and management techniques for fertilizers matched those often advised for the area [95]. Fertilizers were 60 and 30 kg/ha of nitrogen and phosphorus oxide, respectively. Experiments were planted in mid-September in all years and harvested in mid-August. The irrigated foothill zone where KRIAPG is located is a relatively well-watered location; the experimental materials were irrigated 3 times during their development at a rate of 600 m^3^/ha and kept free from weeds.

Weather conditions were more favorable for leaf rust development in 2022 than in 2021 (http://weatherarchive.ru (accessed on 15 April 2023)). In May, the amount of precipitation exceeded the norm, which led to an increase in environmental humidity and contributed to the effective infection of plants with spores of *Puccinia triticina* (Table 4).

Mixed races of *Puccinia triticina* urediniospores identified in 2021 (TJTTR, MKTKQ, TDTTR, TFTTQ and MFTTQ) were used to inoculate field plots in both test years (2021 and 2022). The ratio of the urediospores of the five selected races forming each year’s inoculation was determined according to their frequencies in the previous years (2020). The percentages of the five races used to make the urediniospore mixtures for the 2021–2022 test were TJTTR (30%), MKTKQ (30%), TDTTR (15%), TFTTQ (15%) and MFTTQ (10%). The susceptible cultivar Morocco was used to multiply the inoculum. Plants were inoculated with a mixture of spores and talc (1:100) at a rate of 20 mg spores per 1 m^2^ (5–10 × 10^4^ spores) at the boot stage [91,96]. After spraying the plants with water, the inoculum was applied using the dusting method [91]. The spore concentration was 10 times higher than in experiments with seedlings. After inoculation, the areas with plants were covered with polyethylene film for 16–18 h. The second inoculation was conducted after 10–12 days, when no visible symptoms were observed. Leaf rust severity was recorded on individual plants following the modified Cobb scale [91,97], which includes disease severity (percentage of leaf area covered with rust urediniospores) as well as disease response (infection type). The infection types were recorded as 0—immune (no uredinia or other macroscopic sign of infection); R—resistant (miniature uredinia and spots of chlorosis, occupying up to 5–10% leaf); MR—moderately resistant (small uredinia and chlorotic zones occupying not more than 10–25%); MS—moderately susceptible (small pustules occupying up to 40–50% leaf surface); and S—susceptible (large pustules ranging from 50 to 100% leaf surface). Data recording began with the appearance of the first symptoms in the susceptible control (Morocco). When the plots were in the boot and milk phases, in late May and early June, respectively, infection type and severity data were collected. The second examination began when the level of rust in the susceptible control, Morocco, reached 60 to 80%.

Productivity was characterized by the major components, namely plant height (PH, cm), days to heading (DH), spike lengths (SL, cm), the mean number of spikelets/spike (SS), grains/spike (GS), the weight of grain/spike (WGS, g), and thousand kernel weights (TKW, g). The weight of a thousand kernels was estimated in grams with the measurement of the mass of seeds after adjusting the moisture content to 12% [98]. Given that genotypic variation for NDVI can be used to identify heat-tolerant and high-yielding germplasm, four normalized difference vegetation index (NDVI) measurements were taken using a portable device, GreenSeeker (Trimble Navigation Ltd., Sunnyvale, CA, USA), on 27 May and, 5, 15 and 25 June in 2021 and 2022 when all wheat genotypes were near or at Zadoks growth stages Z49 (booting), Z69 (flowering), Z75 (milk) and Z83 (dough) [96]. NDVI measurements correspond to the same growth stages in 2021 and 2022.

### 4.5. Statistical Data Processing

According to Saari and Wilcoxson (1974), the average Coefficient of Infection (ACI) was determined by multiplying the severity values by the constants for infection types: R (resistant) = 0.2; MR (moderately resistant) = 0.4; MS (moderately susceptible) = 0.8; and S (susceptible) = 1 [99]. The following formula, developed by Wilcoxon et al. [100], was used to determine the area under the disease progress curve (AUDPC):

(1) $$AUDPC=\sum_{i=1}^{n-1}\frac{y_{i}+y_{i+1}}{2}\times (t_{i+1}-t_{i})$$

*y_i_*—an assessment of disease at the *i*th observation;

*t_i_*—time (in days) at the *i*th observation;

*n*—the total number of observations.

The susceptibility index (φ) is calculated from the ratio of the AUDPC of the sample to the AUDPC of the susceptible control.

In order to determine genotypic and year variances among genotypes for traits of productivity and leaf rust resistance, analysis of variance (ANOVA) was performed using R-studio software, and coefficients of Pearson correlation were calculated using the mean values of the characters assessed. Principal component analysis was performed, and biplots were prepared using R-studio software in R version 3.5.3 [101]. The broad-sense heritability index, which measures the percentage of phenotypic variation attributable to genetic determinants, was derived using the ANOVA results *h_b_*^2^ = SS_g_/SS_t_, where SS_g_ is the sum of squares for genotype and SS_t_ is the total sum of squares.

### 4.6. DNA Extraction and Molecular Screening of Lr Resistance Genes

Each genotype’s genomic DNA was extracted using the CTAB method from the fresh leaves of individual plants at the two-leaf seedling stage [102]. The concentration and purity of the resulting preparation were measured using a NanoDrop One spectrophotometer. The DNA concentration for PCR was adjusted to 20 ng/µL. Primers linked to *Lr* genes were employed according to certain approved protocols. The polymerase chain reaction (PCR) was conducted using the primers and annealing temperature settings that were specified for each *Lr* gene in the references (Table 5). A Bio-Rad T100TM Thermal Cycler (Bio-RAD, Hercules, California, USA) was used to conduct the PCR experiments. The PCR mixture (25 µL) contained 2.5 µL of genomic DNA (30 ng), 1 µL of each primer (1 pM/µL) (Sigma-Aldrich, St. Louis, MI, USA), 2.5 µL of dNTP mixture (2.5 mM, dCTP, dGTP, dTTP and dATP aqueous solution) (ZAO Sileks, Russia), 2.5 µL MgCl_2_ (25 mM), 0.2 µL Taq polymerase (5 units µL) (ZAO Sileks, Russia), 2.5 µL 10×PCR buffer and 12.8 µL ddH20. TBE buffer (45 mM Tris-borate, 1 mM EDTA, pH 8) was used to separate the amplification products, and ethidium bromide was added [103]. A 100-bp DNA ladder (Fermentas, Vilnius, Lithuania) was employed to gauge the size of the amplification fragment. The Gel Documentation System (Gel Doc XR+, BIO-RAD, Hercules, CA, USA) was used to visualize the results. Each sample underwent three separate tests.

## 5. Conclusions

In this study, a collection of 70 winter wheat genotypes showed phenotypic diversity in leaf rust resistance. Two virulent races of *Puccinia triticina* were tested. The results indicated a significant positive correlation between seedling resistance and adult plant resistance for 2021 and 2022. A highly significant negative correlation was found between the AUDP and the weight of a thousand kernels in susceptible accessions. Twelve wheat accessions that were resistant both at the seedling and adult plant stages were selected, and they can be used directly in breeding programs to improve the leaf rust resistance of wheat. The molecular screening revealed twenty-seven carriers of a single effective *Lr* resistance gene, ten carriers of two *Lr* genes, six carriers of three *Lr* genes, and two carriers of four *Lr* genes. Large-scale single-gene variety cultivation places pathogens under intense selection pressure, which may eventually cause the establishment of an epiphytotic disease [105,106]. In order to improve the resistance of winter wheat to leaf rust in Central Asia, breeding programs can make use of the carriers of useful *Lr* genes that were discovered in this study.

## Figures and Tables

**Table 1:** Analysis of variance of the effect of plant genotype and pathogen race on the resistance of wheat seedlings to leaf rust.

Factor	SS	df	MS	F-Value	h_b_^2^, %
Genotype	171.84	69	2.49	13.19 ***	0.90
Race	4.46	1	4.46	23.63 ***	
Residuals	13.03	69	0.19		
Total	189.34	139			

Notes: SS—a sum of squares; df—degree of freedom; MS—mean squares; h_b_^2^—broad sense heritability index. *** Significant difference at *p* < 0.001.

**Table 2:** Disease severity for leaf rust and detected *Lr* genes based on linked markers in the collection of winter wheat genotypes.

Cultivar Name	Leaf Rust Severity 2021			AUDPC	φ, %	Leaf Rust Severity 2022			AUDPC	φ, %	Reaction to Infection with Races *P. triticina*		*Lr* Gene (s) Detected Based on Linked Markers
	1st Score	2nd Score	3rd Score			1st Score	2nd Score	3rd Score			MKTKQ	TJTTR	
Alatau	0	5R	10MR	30	3	0	10MR	20MS	120	10	2	3	*Lr46*
Almaly	10MR	15MR	20MR	120	12	10MS	20MS	50S	450	38	2	3	*Lr34*
Aliya	5MR	10MR	20MS	130	13	30MS	50S	70S	970	83	2	2	*Lr10*, *Lr28*, *Lr37*
Azharly	5MR	5MR	30MS	150	15	0	0	10MR	20	2	0	0	*Lr46*
Akbidaj	5MR	15MR	30MS	190	19	10R	30MS	50S	500	43	3	4	*Lr46*
Batyr	0	10MR	30MS	160	16	0	10MR	10MR	60	5	3	3	*Lr46*
Egemen 20	10MR	20MS	30MS	300	29	0	0	0	0	0	2	2	*-*
Farabi	10MR	20MS	30MS	300	29	0	10MS	30MS	200	17	3	3	*Lr46*
Mataj	10MR	30MS	40S	460	45	0	5R	20MS	90	8	2	2	*Lr9*, *Lr37*, *Lr46*
Kyzyl bidaj	0	10MR	30MS	160	16	20MS	30MS	40S	520	44	4	4	*Lr37*, *Lr19*
Keremet	0	0	0	0	0	0	0	0	0	0	0	0	*Lr19*, *Lr26*, *Lr37*, *Lr46*
Koksu	10MR	10MR	30MS	180	18	0	0	10MS	40	3	2	2	*Lr46*
Kokbidaj	5R	5R	20MS	95	9	0	0	10MR	20	2	2	3	*Lr37*
Karaspan	5R	20MS	40S	365	36	20MS	30MS	50S	570	49	4	4	*Lr34*
Karlygash	10MR	10MR	40MS	220	22	0	0	0	0	0	2	3	*Lr34*
Kazakhstanskaya 10	5MR	20MS	30MS	290	28	0	5MR	30MS	140	12	2	3	*-*
Progress	10MS	30MS	50S	530	52	5R	20MS	40S	365	31	4	4	*-*
Prezident	10MS	30MS	30MS	400	39	0	10MS	20MS	160	14	2	3	*-*
Raminal	10R	10MR	30MS	170	17	0	10R	10R	30	3	2	2	*Lr9*, *Lr34*
Rasad	0	0	0	0	0	0	0	0	0	0	1	2	*Lr34*, *Lr37*, *Lr46*
Rausin	20MS	50S	70S	930	91	20MS	40S	40S	680	58	4	4	*-*
Reke	10MR	20MR	30MS	220	22	10MR	20MS	50S	430	37	4	4	*Lr28*, *Lr37*, *Lr46*
Zhalyn	10MS	30MS	50S	530	52	10MR	20MS	30MS	300	26	4	4	*-*
Yuzhnaya 12	0	20MS	40S	360	35	20MS	30MS	50S	570	49	3	4	*Lr9*
Pirotriks 50	10MS	30MS	40MS	440	43	10MS	20MS	40S	400	34	3	4	*Lr28*
Daulet	20MS	60S	60S	980	96	10MS	30MS	50S	530	45	4	4	*-*
Konditerskaya	10MR	10MS	40MS	260	25	0	10MS	30MS	200	17	2	3	*Lr28*
428/MK-122A-1	5MR	10MS	40MS	250	25	0	10MS	30MS	200	17	3	4	*-*
Steklovidnaya 24	0	0	30MS	120	12	0	20MS	30S	310	26	3	2	*-*
Sultan 2	5R	20MS	60S	465	46	0	20MS	40S	360	31	4	4	*-*
Naz/Immun 78	20MS	40S	60S	780	76	20MS	50S	70S	930	79	4	4	*-*
Naz/GF 55-2	5R	5R	5R	20	2	0	0	0	0	0	2	1	*Lr34*
Naz/GF 55-3	0	10MR	30MS	160	16	10MR	30MS	50S	510	44	4	3	*Lr26*, *Lr68*
Naz/GF 55-5	0	20MS	30MS	280	27	10MR	40S	40S	620	53	3	3	*-*
Yr/Octyabrina	10MR	30MS	40MS	420	41	20MS	20MS	50S	490	42	4	4	*Lr10*
425/Obri	0	10MR	40MS	200	20	10MR	30MS	50S	510	44	3	4	*Lr28*
Alihan	0	0	0	0	0	0	0	0	0	0	1	2	*Lr37*, *Lr68*
Anar	0	5R	30MS	130	13	0	10R	30MS	140	12	2	3	*Lr9*
Derbes	20MS	40S	50S	730	72	20MS	40S	40S	680	58	4	4	*-*
Krasnovodapadskaya 210	0	30MS	40MS	400	39	10MS	30MS	30S	430	37	3	4	*Lr37*
Almatinskaya polukarlikovaya/Progress	10MS	30MS	30MS	400	39	0	0	10R	10	1	2	2	*-*
Almaly/Orbij	5MR	10MS	30MS	210	21	0	0	0	0	0	1	2	*Lr9*, *Lr34*, *Lr68*
Gozgon	0	0	5R	5	0	0	0	0	0	0	2	2	*-*
Bunyodkor	0	20MS	30S	310	30	10MR	20MS	30S	330	28	1	1	*Lr26*
Faravon	0	5MR	30MS	140	14	0	0	5MR	10	1	2	3	*-*
Hazrati Bashir	15MR	30MS	40S	470	46	5MR	20MS	40S	370	32	3	3	*Lr10*
Hisorok	0	0	0	0	0	0	0	0	0	0	1	1	*Lr9*, *Lr10*, *Lr19*, *Lr37*
Layagatlii 80	0	10MS	30MS	200	20	0	0	0	0	0	1	1	*Lr10*, *Lr26*
Shafag 2	0	10MS	60S	380	37	0	10MS	30MS	200	17	0	0	*Lr26*
Egana	0	0	0	0	0	0	0	0	0	0	2	1	*Lr9*, *Lr10*, *Lr34*
415-SP-2	0	5MR	5MR	21	2	0	0	10MR	14	1	2	2	*-*
416-SP-2	0	0	0	0	0	0	0	0	0	0	1	2	*Lr34*, *Lr26*
2-CP	0	10MR	20MS	84	8	10MR	10MS	30MS	154	13	4	4	*Lr34*
3-CP	0	0	20MS	56	5	0	10MR	20MS	84	7	3	4	*Lr37*
4-CP	0	10MS	10MS	84	8	10MR	30MS	30MS	266	23	3	3	*Lr34*
5-CP	0	5R	10MS	35	3	0	20MS	30MS	196	17	3	3	*Lr34*
6-CP	0	10MS	10MS	84	8	10MR	10MS	20MS	126	11	1	2	*-*
7-CP	0	0	0	0	0	0	0	0	0	0	0	1	*Lr37*
8-CP	0	20MS	30S	217	21	20MS	40S	60S	546	47	3	4	*-*
9-CP	10MS	10MS	30MS	168	16	0	0	0	0	0	1	2	*Lr26*, *Lr37*
10-CP	0	0	10MS	28	3	0	0	0	0	0	2	3	*-*
11-CP	0	20MS	30MS	196	19	10MS	10MS	30MS	168	14	4	4	*Lr37*
12-CP	0	0	0	0	0	0	20MS	30MS	196	17	3	3	*-*
13-CP	0	0	0	0	0	0	0	0	0	0	0	2	*-*
14-CP	0	10MS	20MS	112	11	0	10MR	10MS	56	5	2	2	*Lr26*, *Lr37*
15-CP	0	0	10MS	28	3	0	20MS	40MS	224	19	3	3	*Lr34*, *Lr37*
16-CP	0	5R	10MS	35	3	10MS	30MS	40MS	308	26	3	4	*Lr34*
17-CP	0	0	0	0	0	0	10MR	20MR	56	5	2	2	*Lr34*, *Lr37*
18-CP	10MS	40S	50S	483	47	20MS	50S	50S	581	50	4	4	*-*
19-CP	10MS	10MS	30MS	168	16	10MS	20MS	40MS	252	22	3	4	*Lr34*
**Controls**													
Morocco	30MS	50S	80S	1020	100	30MS	60S	90S	1170	100			*-*
Transfer/6*TC	0	0	0	0	0	0	0	10MR	20	2			*Lr9*
TC*6/Exchange	0	20MS	40MS	320	31	0	5R	20MS	90	8			*Lr10*
TC*7/Tr	0	0	0	0	0	0	0	0	0	0			*Lr19*
TC*6/ST-1-25	0	10MR	30MS	160	16	0	0	20MS	80	7			*Lr26*
CS2D-2M	0	0	0	0	0	0	0	0	0	0			*Lr28*
TC*6/PI58548	0	0	20MS	80	8	0	10MR	20MS	120	10			*Lr34*
TC*6/VPM	0	20MS	30MS	280	27	0	0	10MR	20	2			*Lr37*
Pavon 76	10MR	20MS	40MS	340	33	0	20MS	30MS	280	24			*Lr46*
Parula	0	0	10MR	20	2	0	0	0	0	0			*Lr68*

Note: φ—measure of susceptibility index; AUDPC—the area under the disease progress curve; TJTTR and MKTKQ—*Puccinia triticina* races.

**Table 3:** Virulence characterization of the *P. triticina* races used in the study.

Race	Virulence Formula (Avirulent/Virulent)	Response of *Lr* Genes (%)	
		R	S
TJTTR	*Lr9*, *Lr19*, *Lr26*/*Lr1*, *Lr2a*, *Lr2c*, *Lr3*, *Lr16*, *Lr24*, *Lr3ka*, *Lr11*, *Lr17*, *Lr30*, *Lr2b*, *Lr3bg*, *Lr14a*, *Lr14b*, *Lr15*, *Lr18*, *Lr20*	15	85
MKTKQ	*Lr2a*, *Lr2b*, *Lr2c*, *Lr9*, *Lr19*, *Lr20*/*Lr1*, *Lr3*, *Lr16*, *Lr24*, *Lr26*, *Lr3ka*, *Lr11*, *Lr17*, *Lr30*, *Lr3bg*, *Lr14a*, *Lr14b*, *Lr15*, *Lr18*	30	70

**Table 4:** Meteorological data on average temperature and precipitation for the growing season in the fields of KazNIIZiR for 2021–2022.

Year	Month	Temperature (°C)	Monthly Rainfalls (mm)	Average Relative Humidity (%)
2021	April	12.5	54	50
	May	19.5	70	51
	June	23.0	20	38
2022	April	16.7	45	54
	May	19.0	142	65
	June	24.3	36	49

**Table 5:** Molecular markers used to identify *Lr* genes.

Gen	Chr	Type of Marker	Primer Name	Sequence of Primers 5′-3′	Anneling t°C	Fragmet Size, b.p.	Reference
*Lr9*	6BL	STS	J13-1	5′-CCACACTACCCCAAAGAGACG-3′	62	1100	[51]
			J13-2	5′-TCCTTTTATTCCGCACGCCGG-3′			
*Lr10*	1AS	STS	F1.2245	5′-GTGTAATGCATGCAGGTTCC-3′	57	310	[104]
			Lr10-6/r2	5′-AGGTGTGAGTGAGTTATGTT-3′			
*Lr19*	7AL	STS	Psy1-EF2	5′-CAAGTTCCCCATAGATATTCAG-3′	63	191	[53]
			Psy1-ER4	5′-AGAGAAAACCATTGCATCTGTA-3′			
*Lr26*	1BL	STS	Iag 95	5′-CTCTGTGGATAGTTACTTGATCGA-3′ 5′-CCTAGAACATGCATGGCTGTTACA-3′	55	1100	[55]
*Lr28*	4AL	SSR	WMC 313	5′-CCCGGCATAAGTCTATGGTT-3 5′-CAATGAATGAGATACGTGAA-3′	51	320	[56]
*Lr34*	7DS	STS	csLV34	5′-GTTGGTTAAGACTGGTGATGG-3′ 5′-TGCTTGCTATTGCTGAATAGT-3′	55	+150	[43]
						−229	
*Lr37*	2AS	CAPS	Uric	5′-GGTCGCCCTGGCTTGCACCT-3′	64	285	[44]
			Ln2	5′-TGCAGCTACAGCAGTATGTACACAAAA-3′			
*Lr46*	1BL	SSR	Wmc44	5′-GGT CTT CTG GGC TTT GAT CCT G-3′ 5′-GTT GCT AGG GAC CCG TAG TGG-3′	61	242	[58]
*Lr68*	7BL	STS	csGS	5′-AAG ATT GTT CAC AGA TCC ATG TCA-3′ 5′-GAG TAT TCC GGC TCA AAA AGG-3′	60	385	[60]

## Data Availability

The data presented in this study are available on request from the corresponding author.

## Supplementary Materials

The following supporting information can be downloaded at: https://www.mdpi.com/article/10.3390/plants12152786/s1, Figure S1: Pearson correlation analysis between the disease progression value (AUDPC) and the main indicators of wheat productivity in 2021 (**a**) and 2022 (**b**); Figure S2: DNA amplification products of wheat accessions using primers to the STS J13 locus linked with the *Lr9* resistance gene; Figure S3: DNA amplification products of wheat accessions using primers to the STS *Lr10* locus linked with the *Lr10* resistance gene; Figure S4: DNA amplification products of wheat accessions using primers to the STS PSY_EF2/PSY_ER4 locus linked with the *Lr19* resistance gene; Figure S5: DNA amplification products of wheat accessions using primers to the STS Iag 95 locus linked with the *Lr26* resistance gene; Figure S6: DNA amplification products of wheat accessions using primers to the SSR wmc313 locus linked with the *Lr28* resistance gene; Figure S7: DNA amplification products of wheat accessions using primers to the STS csLV34 locus linked with the *Lr34* resistance gene; Figure S8: DNA amplification products of wheat accessions using primers to the CAPS URIC-LN2 linked with the *Lr37* resistance gene; Figure S9: DNA amplification products of wheat accessions using primers to the STS Xwmc 44 locus linked with the *Lr46* resistance gene; Figure S10: DNA amplification products of wheat accessions using primers to the STS csGS F1 csGS R-1 locus linked with the *Lr68* resistance gene; Table S1: Analysis of variance (ANOVA) on traits of productivity and leaf rust resistance of a winter wheat collection; Table S2: Agronomic performance of the 70 wheat cultivars and breeding lines evaluated at the KRIAPG station, Kazakhstan; Table S3: Pedigree of perspective winter wheat lines.

## References

[1] Food and Agriculture (FAO) Organization of the United Nation. http://www.fao.org/faostat/en/#data/CC.

[2] Statista. https://www.statista.com/.

[3] Statistics Committee Ministry of National Economy of the Republic of Kazakhstan. https://new.stat.gov.kz/.

[4] Kolmer J. (2013). Leaf Rust of Wheat: Pathogen Biology, Variation and Host Resistance. Forests.

[5] Zhao J., Wang M., Chen X., Kang Z. (2016). Role of Alternate Hosts in Epidemiology and Pathogen Variation of Cereal Rusts. Annu. Rev. Phytopathol..

[6] Bolton M.D., Kolmer J.A., Garvin D.F. (2008). Wheat leaf rust caused by *Puccinia triticina*. Mol. Plant Pathol..

[7] Dinh H.X., Singh D., Periyannan S., Park R.F., Pourkheirandish M. (2020). Molecular genetics of leaf rust resistance in wheat and barley. Theor. Appl. Genet..

[8] Huerta-Espino J., Singh R.P., German S. (2011). Global status of wheat leaf rust caused by *Puccinia triticina*. Euphytica.

[9] Pretorius Z.A., Visser B., Terefe T. (2015). Races of *Puccinia triticina* detected on wheat in Zimbabwe, Zambia and Malawi and regional germplasm responses. Australas. Plant Pathol..

[10] Roelfs A.P. (1992). Barley stripe rust in Texas. Plant Dis..

[11] Singh R.P., Huerta-Espino J., Pfeiffer W., Figueroa-Lopez P. (2004). Occurrence and impact of a new leaf rust race on durum wheat in northwestern Mexico from 2001 to 2003. Plant Dis..

[12] Riaz M., Wong Y. (2017). Estimation of Yield Losses Due to Leaf Rust and Late Seeding on Wheat (*Triticum aestivum* L.) Variety Seher-06 in District Faisalabad, Punjab, Pakistan. Adv. Biotech. Micro..

[13] Koyshibaev M.K. (2018). Diseases of Wheat.

[14] Keishilov Z., Kokhmetova A., Kumarbaeva M., Bolatbekova A., Malysheva A., Kokhmetova A. (2022). Monitoring of leaf rust (Puccinia recondita) of spring wheat in Northern Kazakhstan 2019–2022. Herald Sci. KazATU.

[15] Morgounov A., Akin B., Demir L., Keser M., Kokhmetova A., Martynov S., Yessimbekova M. (2015). Yield gain due to fungicide application in varieties of winter wheat (*Triticum aestivum*) resistant and susceptible to leaf rust. Crop Pasture Sci..

[16] Galymbek K., Kokhmetova A.M., Akan K., Madenova A.K., Atishova M.N. (2017). Identification of germplasm of Wheat on leaf rust (Puccinia recondita Rob. Ex Desm. F.sp. Tritici). Ecol. Environ. Conserv..

[17] Kokhmetova A., Rsaliyev S., Atishova M., Kumarbayeva M., Malysheva A., Keishilov Z., Zhanuzak D., Bolatbekova A. (2021). Evaluation of wheat germplasm for resistance to leaf rust (*Puccinia triticina*) and Identification of the Sources of Lr Resistance Genes Using Molecular Markers. Plants.

[18] Kokhmetova A., Atishova M., Madenova A., Kumarbayeva M. (2019). Genotyping of wheat germplasm for resistance to toxins of tan spot Pyrenophora tritici-repentis. J. Biotechnol..

[19] Kokhmetova A., Kovalenko N., Kumarbaeva M. (2020). Pyrenophora tritici-repentis population structure in the Republic of Kazakhstan and identification of wheat germplasm resistant to tan spot. Vavilov J. Genet. Breed..

[20] Kokhmetova A., Kumarbayeva M., Atishova M., Nehe A., Riley I., Morgounov A. (2021). Identification of high-yielding wheat genotypes resistant to Pyrenophora tritici-repentis (tan spot). Euphytica.

[21] Kumarbayeva M., Kokhmetova A., Kovalenko N., Atishova M., Keishilov Z., Aitymbetova K. (2022). Characterization of Pyrenophora tritici-repentis (tan spot of wheat) races in Kazakhstan. Phytopathol. Mediterr..

[22] Kokhmetova A., Sharma R., Rsaliyev S., Galymbek K., Baymagambetova K., Ziyaev Z., Morgounov A. (2018). Evaluation of Central Asian wheat germplasm for stripe rust resistance. Plant Genet. Resour..

[23] Kokhmetova A., Rsaliyev A., Malysheva A., Atishova M., Kumarbayeva M., Keishilov Z. (2021). Identification of stripe rust resistance genes in common wheat cultivars and breeding lines from Kazakhstan. Plants.

[24] Malysheva A., Kokhmetova A., Kumarbayeva M., Zhanuzak D., Bolatbekova A., Keishilov Z., Gultyaeva E., Kokhmetova A., Tsygankov V., Dutbayev Y. (2022). Identification of Carriers of Puccinia Striiformis Resistance Genes in the Population of Recombinant Inbred Wheat Lines. Int. J. Biol. Chem..

[25] Yessenbekova G., Kokhmetova A., Madenova A., Amanov O., Dutbayev Y., Kampitova G. Identification of Lr34/Yr18 Gene in Wheat Germplasm in Kazakhstan. Proceedings of the 2014 APS-CPS Joint Meeting.

[26] Kokhmetova A.M., Atishova M.N. (2012). Identification of sources of resistance to wheat stem rust using molecular markers. Russ. J. Genet. Appl. Res..

[27] Olivera F.P., Szabo L., Kokhmetova A., Morgunov A., Luster D.G., Jin Y. (2022). Puccinia graminis f. sp. tritici population causing recent wheat stem rust epidemics in Kazakhstan is highly diverse and includes novel virulences. Phytopathology.

[28] Flor H. (1946). Genetics of Pathogenicity in Melampsoralini. J. Agric. Res..

[29] McCallum B., Hiebert C., Cloutier S. (2016). A review of wheat leaf rust research and the development of resistant cultivars in Canada. Can. J. Plant Pathol..

[30] Park R., Golegaonkar P., Derevnina L., Sandhu K., Karaoglu H., Elmansour H., Dracatos P., Singh D. (2015). Leaf rust of cultivated barley: Pathology and control. Annu. Rev. Phytopathol..

[31] Chaves M., Martinelli J., Guterres C., Sganzerla F. (2008). The Cereal Rust: An Overview. Pest Tech..

[32] McIntosh R., Dubcovsky J., Rogers W., Xia X., Raupp W. (2020). Catalogue of Gene Symbols for Wheat: 2020 Supplement. Ann. Wheat Newslett..

[33] Sears E. (1956). The transfer of leaf rust resistance from *Aegilops umbellulata* into wheat. Brookhaven Symp. Biol..

[34] Riley R., Chapman V., Johnson R. (1968). The incorporation of yellow rust resistance of Aegilops comosa into wheat by genetically induced homoeologous recombination. Nature.

[35] Ellis J.G., Lagudah E.S., Spielmeyer W., Dodds P.N. (2014). The past, present and future of breeding rust resistant wheat. Front. Plant Sci..

[36] Parlevliet J., Ommeren A. (1975). Partial resistance of barley to leaf rust, Puccinia hordei. II. Relationship between field trials, micro plot tests and latent period. Euphytica.

[37] Dodds P.N., Rathjen J.P. (2010). Plant immunity: Towards an integrated view of plant–pathogen interactions. Nat. Rev. Genet..

[38] Dyck P., Kerber E., Roelfs A., Bushnell W.R. (1985). Resistance of the Race-Specific Type. The Cereal Rusts.

[39] Park R., McIntosh R. (1994). Adult plant resistances to Puccinia recondita f. sp. tritici in wheat. N. Z. J. Crop Hortic. Sci..

[40] Kolmer J., Singh R., Garvin D., Viccars L., William H., Huerta-Espino J., Obonnaya F., Raman H., Orford S., Bariana H. (2008). Analysis of the Lr34/Yr18 rust resistance region in wheat germplasm. Crop Sci..

[41] Dyck P. (1987). The association of a gene for leaf rust resistance with the chromosome 7D suppressor of stem rust resistance in common wheat. Genome.

[42] Krattinger S., Lagudah E., Spielmeyer W., Singh R., Huerta-Espino J., McFadden H., Bossolini E., Selter L., Keller B. (2009). A putative ABC transporter confers durable resistance to multiple fungal pathogens in wheat. Science.

[43] Lagudah E., McFadden H., Singh R., Huerta-Espino J., Bariana H., Spielmeyer W. (2006). Molecular genetic characterization of the Lr34/Yr18 slow rusting resistance gene region in wheat. Theor. Appl. Genet..

[44] Helguera M., Khan I., Kolmer J., Lijavetzky D., Zhong-Qi L., Dubcovsky J. (2003). PCR Assays for the Cluster of Rust Resistance Genes and Their Use to Develop Isogenic Hard Red Spring Wheat Lines. Crop Sci..

[45] Moore J., Herrera-Foessel S., Lan C., Schnippenkoetter W., Ayliffe M., Huerta-Espino J., Lagudah E. (2015). A recently evolved hexose transporter variant confers resistance to multiple pathogens in wheat. Nat. Genet..

[46] Kumar S., Bhardwaj S., Gangwar O. (2022). Characterization of five new pathotypes of *Puccinia triticina* identified from Northeast India, Nepal, and Bangladesh. Aust. Plant Pathol..

[47] Melchinger A. (1990). Use of molecular markers in breeding for oligogenic disease resistance. Plant Breed..

[48] Singh A., Pallavi J., Gupta P., Prabhu K. (2010). Identification of microsatellite markers linked to leaf rust adult plant resistance (APR) gene 48 in wheat. Plant Breed..

[49] Yue Z., Zai-Feng L., Xing L., Long W., Ye Z., Da-Qun L. (2010). Molecular mapping for leaf rust resistance genes in wheat line Tian 95HF2. Acta Agron. Sin..

[50] Gultyaeva E., Kokhmetova A., Shreyder E., Shaydayuk E., Atishova M., Madenova A., Malysheva A., Galymbek K. (2020). Genetic variability of perspective breeding material of spring bread wheat for resistance to leaf rust in Russia and Kazakhstan. Bull. NAS RK.

[51] Schachermayr G., Siedler H., Gale M., Winzeler H., Winzeler M., Keller B. (1994). Identification and localization of molecular markers linked to the Lr9 leaf rust resistance gene of wheat. Theor. Appl. Genet..

[52] Chelkowski J., Golka L., Stepien L. (2003). Application of STS markers for leaf rust resistance genes in near–isogenic lines of spring wheat cv. Thatcher. J. Appl. Genet..

[53] Zhang W., Dubcovsky J. (2008). Association between allelic variation at the Phytoene synthase 1 gene and yellow pigment content in the wheat grain. Theor. Appl. Genet..

[54] Mago R., Spielmeyer W., Lawrence G. (2002). Identification and mapping of molecular markers linked to rust resistance genes located on chromosome 1RS of rye using wheat-rye translocation lines. Theor. Appl. Genet..

[55] Mago R., Miah H., Lawrence G.J., Wellings C.R., Spielmeyer W., Bariana H.S., Ellis J.G. (2005). High-resolution mapping and mutation analysis separate the rust resistance genes Sr31, Lr26, and Yr9 on the short arm of rye chromosome 1. Theor. Appl. Genet..

[56] Vikal Y., Chhuneja P., Singh R., Dhaliwal H. (2004). Tagging of an *Aegilops speltoides* Derived Leaf Rust Resistance Gene Lr28 with a Microsatellite Marker in Wheat. J. Plant Biochem. Biotechnol..

[57] McIntosh R., Wellings C., Park R. (1995). Wheat Rusts: An Atlas of Resistance Genes.

[58] William M., Singh R., Huerta-Espino J., Islas S., Hoisington D. (2003). Molecular marker mapping of leaf rust resistance gene Lr46 and its association with stripe rust resistance gene Yr29 in wheat. Phytopathology.

[59] Tomkowiak A., Skowrońska R., Kwiatek M., Spychała J., Weigt D., Kurasiak-Popowska D., Niemann J., Mikołajczyk S., Nawracała J., Kowalczewski P. (2021). Identification of leaf rust resistance genes Lr34 and Lr46 in common wheat (*Triticum aestivum* L. ssp. aestivum) lines of different origin using multiplex PCR. Open Life Sci..

[60] Herrera-Foessel S., Singh R., Huerta-Espino J., Rosewarne G., Periyannan S., Viccars L., Calvo-Salazar V., Lan C., Lagudah E. (2012). Lr68: A new gene conferring slow rusting resistance to leaf rust in wheat. Theor. Appl. Genet..

[61] Wellings C. (2011). Global status of stripe rust: A review of historical and current threats. Euphytica.

[62] Morgounov A., Rosseeva L., Koyshibayev M. (2007). Leaf rust of spring wheat in Northern Kazakhstan and Siberia: Incidence, virulence, and breeding for resistance. Aust. J. Agric. Res..

[63] Gultyaeva E., Kovalenko N., Shamanin V., Tyunin V., Shreyder E., Shaydayuk E., Morgunov A. (2018). Population structure of leaf pathogens of common spring wheat in the West Asian regions of Russia and North Kazakhstan in 2017. Vavilov J. Genet. Breed..

[64] Gultyaeva E., Shaydayuk E., Shamanin V., Akhmetova A., Tyunin V., Shreyder E., Kashina I., Eroshenko L., Sereda G., Morgunov A. (2018). Genetic structure of Russian and Kazakhstani leaf rust causative agent *Puccinia triticina* Erikss. populations as assessed by virulence profiles and SSR markers. Agric. Biol..

[65] Gultyaeva E., Shaydayuk E., Kazartsev I., Akhmetova A., Kosman E. (2018). Microsatellite analysis of *Puccinia triticina* from Triticum and Aegilops hosts. Aust. Plant. Path..

[66] Gad M., Hao-xing L., Ming-ju L., El-Orabey W., Hasan M. (2019). Evaluation of wheat genotypes to rust diseases (Puccinia spp.) under agroclimatic conditions of Egypt and China. J. Agric. Crop Res..

[67] Yan X., Li Z., Yang H., Zhang H., Gebrewahid T., Yao Z., Li D., Zhou Y. (2017). Analysis of Wheat Leaf Rust Resistance genes in 30 Important Wheat Cultivars. Sci. Agric. Sin..

[68] Kokhmetova A.M., Atishova M.N., Galymbek K. (2020). Identification of wheat germplasm resistant to leaf, stripe and stem rust using molecular markers. Bull. NAS RK.

[69] Mallick N., Jha S.K., Agarwal P., Mall A., M. N., Kumar S., Choudhary M.K., Bansal S., Saharan M.S., Sharma J.B. (2022). Marker-Assisted Improvement of Bread Wheat Variety HD2967 for Leaf and Stripe Rust Resistance. Plants.

[70] Hanzalová A., Zelba O. (2022). Leaf rust (*Puccinia triticina* Eriks) resistance genes in wheat cultivars registered in the Czech Republic. J. Plant Dis. Prot..

[71] Liu Y., Gebrewahid T., Zhang P., Li Z., Liu D. (2021). Identification of leaf rust resistance genes in common wheat varieties from China and foreign countries. J. Integr. Agric..

[72] Gao P., Zhou Y., Gebrewahid T., Zhang P., Yan X., Li X., Yao Z., Li Z., Liu D. (2019). Identification of known leaf rust resistance genes in common wheat cultivars from Sichuan province in China. Crop Prot..

[73] Liu D., Yuan C., Singh R., Randhawa M., Bhavani S., Kumar U., Huerta-Espino J., Lagudah E., Lan C. (2022). Stripe rust and leaf rust resistance in CIMMYT wheat line “Mucuy” is conferred by combinations of race-specific and adult-plant resistance loci. Front. Plant Sci..

[74] Martinez F., Niks R., Singh R., Rubiales D. (2001). Characterization of Lr46, a gene conferring partial resistance to wheat leaf rust. Hereditas.

[75] Zhang P., Qi A., Zhou Y., Xia X., He Z., Li Z., Liu D. (2017). Quantitative trait loci mapping of adult-plant resistance to leaf rust in a Fundulea 900 × ‘Thatcher’ wheat cross. Plant Breed..

[76] Lagudah E., Krattinger S., Herrera-Foessel S., Singh R., Huerta-Espino J., Spielmeyer W., Brown-Guedira G., Selter L., Keller B. (2009). Gene-specific markers for the wheat gene Lr34/Yr18/Pm38 which confers resistance to multiple fungal pathogens. Theor. Appl. Genet..

[77] Pal D., Bhardwaj S., Sharma P., Sharma D., Khan H., Prabhu K. (2020). Molecular marker aided selection for developing rust resistant genotypes by pyramiding Lr19/Sr25 and Yr15 in wheat (*Triticum aestivum* L.). Australas. Plant Pathol..

[78] Zhang P., Gebrewahid T., Zhou Y., Li Q., Li Z., Liu D. (2019). Seedling and adult plant resistance to leaf rust in 46 Chinese bread wheat landraces and 39 wheat lines with known Lr genes. J. Integr. Agric..

[79] Kumar K., Jan I., Saripalli G., Sharma P., Mir R., Balyan H., Gupta P. (2022). An update on resistance genes and their use in the development of leaf rust-resistant cultivars in wheat. Front. Genet..

[80] Hanzalová A., Šliková S., Hudcovicová M. (2022). Virulence of wheat leaf rust (*Puccinia triticina* Eriks.) and Lr resistance genes in wheat cultivars in the Slovak Republic in the years 2016–2019. Cereal Res. Commun..

[81] Nemati Z., Mostowfizadeh-Ghalamfarsa R., Dadkhodaie A., Mehrabi R., Steffenson B. (2020). Virulence of Leaf Rust Physiological Races in Iran from 2010 to 2017. Plant Dis..

[82] Goyeau H., Berder J., Czerepak C., Gautier A., Lanen C., Lannou C. (2012). Low diversity and fast evolution in the population of *Puccinia triticina* causing durum wheat leaf rust in France from 1999 to 2009, as revealed by an adapted differential set. Plant Pathol..

[83] Imbaby I., Mahmoud M., Hassan M., Abd-El-Aziz A. (2014). Identification of Leaf Rust Resistance Genes in Selected Egyptian Wheat Cultivars by Molecular Markers. Sci. World J..

[84] Gultyaeva E., Gannibal P., Shaydayuk E. (2023). Long-Term Studies of Wheat Leaf Rust in the North-Western Region of Russia. Agriculture.

[85] Ivanova V. (2022). Physiological specialization of wheat leaf rust (*Puccinia triticina* Eriks.) in Bulgaria. Rom. Agric. Res..

[86] Kolmer J., Hughes M. (2018). Physiologic Specialization of *Puccinia triticina* on wheat in the United States in 2016. Plant Dis..

[87] Tomkowiak A., Bobrowska R., Kwiatek M., Spychala J., Kuczynski J., Tyczewska A., Kowalczewski P., Weigt D., Kosiada T. (2023). Analysis of miRNA expression associated with gene Lr34 responsible for resistance mechanisms to wheat leaf rust. Pak. J. Bot..

[88] Tomkowiak A., Jędrzejewski T., Spychała J., Kuczyński J., Kwiatek M., Tyczewska A., Skowrońska R., Twardowski T. (2020). Analysis of miRNA expression associated with the Lr46 gene responsible for APR resistance in wheat (*Triticum aestivum* L.). J. Appl. Genet..

[89] Mikhailova L.A., Gultyaeva E.I., Mironenko N.V. (1998). Methods for studying the structure of populations of the leaf rust causative agent. Collection of Guidelines on Plant Protection.

[90] Lind V., Gultyaeva E. (2007). Virulence of *Puccinia triticina* on winter wheat in Germany and the European regions of Russian Federation. J. Phytopathol..

[91] Roelfs A., Singh R., Saari E. (1992). Rust Diseases of Wheat: Concept and Methods of Disease Management.

[92] Long D., Kolmer J. (1989). A North American System of Nomenclature for *Puccinia triticina*. Phytopathology.

[93] Gultyaeva E., Shaydayuk E., Kosman E. (2020). Regional and temporal differentiation of virulence phenotypes of *Puccinia triticina* from common wheat in Russia during the period 2001–2018. Plant. Pathol..

[94] Mains E., Jackson H. (1926). Physiologic specialization in the leaf rust of wheat, *Puccinia triticina*. Phytopathology.

[95] Dospekhov B.A. (1985). Methods of Field Experience (With the Basics of Statistical Processing of Research Results).

[96] Zadoks J.C., Chang T.T., Konzak C.F. (1974). A decimal code for the growth stages of cereals. Weed Res..

[97] Peterson R.F., Campbell A., Hannah A. (1948). A Diagrammatic Scale for Estimating Rust Intensity on Leaves and Stems of Cereals. Can. J. Res..

[98] AACC (American Association of Cereal Chemists) (2000). International Approved Methods of the American Association of Cereal Chemists.

[99] Saari E., Wilcoxson R. (1974). Plant disease situation of high-yielding durum wheat in Asia and Africa. Annu. Rev. Phytopathol..

[100] Wilcoxson R., Skovmand B., Atif A. (1974). Evaluation of wheat cultivars ability to retard development of stem rust. Ann Appl Biol..

[101] R Core Team (2018). R: A Language and Environment for Statistical Computing.

[102] Riede C., Anderson J. (1996). Linkage of RFLP markers to an aluminum tolerance gene in wheat. Crop Sci..

[103] Chen X., Line R., Leung H. (1998). Genome scanning for resistance-gene analogs in rice, barley, and wheat by high-resolution electrophoresis. Theor. Appl. Genet..

[104] Schachermayr G., Feuillet C., Keller B. (1997). Molecular markers for the detection of the wheat leaf rust resistance gene Lr10 in diverse genetic backgrounds. Mol. Breed..

[105] Wu H., Kang Z., Li X., Li Y., Li Y., Wang S., Liu D. (2020). Identification of Wheat Leaf Rust Resistance Genes in Chinese Wheat Cultivars and the Improved Germplasms. Plant Dis..

[106] Zhang P., Yan X., Gebrewahid T., Zhou Y., Yang E., Xia X., He Z., Li Z., Liu D. (2021). Genome-wide association mapping of leaf rust and stripe rust resistance in wheat accessions using the 90K SNP array. Theor. Appl. Genet..

