# Supplementary material for: Phenotyping and Identification of Molecular Markers Associated with Leaf Rust Resistance in the Wheat Germplasm from Kazakhstan, CIMMYT and ICARDA
Source: Plants (Basel). 2023 Jul 27;12(15):2786. doi: 10.3390/plants12152786 (PMC10421303; doi:10.3390/plants12152786)
Supplement: Supplementary file 1 [file plants-12-02786-s001.zip › plants-2443329-supplementary.pdf]

plants

*Supplementary materials*

# Phenotyping and Identification of Molecular Markers Associated with Leaf Rust Resistance in the Wheat Germplasm from Kazakhstan, CIMMYT and ICARDA

**Angelina Malysheva [1,*], Alma Kokhmetova [1,*], Rakhym Urazaliev [2], Madina Kumarbayeva [1], Zhenis Keishilov [1], Makpal Nurzhuma [1], Ardak Bolatbekova [1] and Assiya Kokhmetova [1]**

**Table S1.** Analysis of variance (ANOVA) on traits of productivity and leaf rust resistance of a winter wheat collection

| Experiment | Factor | SS | df | MS | F-value | p-Value | $h_b^2$, % |
|---|---|---|---|---|---|---|---|
| AUDPC | Genotype | 5923734 | 69 | 85851 | 3.89 | <0.001 | 0.79 |
| | Year | 5197 | 1 | 5197 | 0.235 | 0.62 | |
| | Residuals | 1522749 | 69 | 22069 | | | |
| NDVI | Genotype | 0.3701 | 69 | 0.005363 | 1.486 | 0.05 | 0.59 |
| | Year | 0.009 | 1 | 0.009446 | 2.617 | 0.11 | |
| | Residuals | 0.2491 | 69 | 0.003610 | | | |
| DH | Genotype | 1277.89 | 69 | 18.52 | 2.758 | <0.001 | 0.43 |
| | Year | 1230.18 | 1 | 1230.1 | 183.2 | <0.001 | |
| | Residuals | 463.32 | 69 | 6.71 | | | |
| PH | Genotype | 27987.9 | 69 | 405.62 | 3.19 | <0.001 | 0.66 |
| | Year | 5136.46 | 1 | 5136.46 | 40.4 | <0.001 | |
| | Residuals | 8773.54 | 69 | 127.15 | | | |
| SL | Genotype | 181.83 | 69 | 2.635 | 3.192 | <0.001 | 0.75 |
| | Year | 1.45 | 1 | 1.454 | 1.762 | 0.18 | |
| | Residuals | 56.95 | 69 | 0.825 | | | |
| SS | Genotype | 390.18 | 69 | 5.654 | 1.994 | <0.01 | 0.64 |
| | Year | 18.84 | 1 | 18.841 | 6.64 | 0.012 | |
| | Residuals | 195.71 | 69 | 2.836 | | | |
| GS | Genotype | 4808.48 | 69 | 69.68 | 1.63 | <0.05 | 0.57 |
| | Year | 549.96 | 1 | 549.96 | 12.94 | <0.001 | |
| | Residuals | 2933.25 | 69 | 42.51 | | | |
| WGS | Genotype | 12.68 | 69 | 0.183 | 1.414 | 0.076 | 0.56 |
| | Year | 1.02 | 1 | 1.028 | 7.91 | <0.01 | |
| | Residuals | 8.97 | 69 | 0.13 | | | |
| TKW | Genotype | 2320.07 | 69 | 33.62 | 2.91 | <0.001 | 0.74 |
| | Year | 14.43 | 1 | 14.43 | 1.24 | 0.26 | |
| | Residuals | 797.07 | 69 | 11.55 | | | |

Note: PH, cm - plant height, DH - days to heading, SL, cm - spike lengths, SS - the mean number of spikelets/spike, GS - grains/spike, WGS, g - the weight of grain/spike, TKW, g - thousand kernel weights

**Table S2.** Agronomic performance of the 70 wheat cultivars and breeding lines evaluated at the KRIAPG station, Kazakhstan. Data are the means of two assessments made in 2021 and 2022.

| # | Name | Origin | 2021 | | | | | | | | 2022 | | | | | | | |
|---|---|---|---|---|---|---|---|---|---|---|---|---|---|---|---|---|---|---|
| | | | NDVI | DH | PH, cm | SL, cm | SS | GS | WGS, g | TKW, g | NDVI | DH | PH, cm | SL, cm | SS | GS | WGS, g | TKW, g |
| 1 | Alatau | KZ | 0.73 | 227 | 110 | 11.73±1.13 | 19.71±1.70 | 42.60±4.27 | 1.86±0.29 | 43.62 | 0.72 | 217 | 125 | 10.54±0.78 | 19.8±1.62 | 46.4±4.33 | 2.31±0.35 | 49.78 |
| 2 | Almaly | KZ | 0.71 | 232 | 78 | 8.53±0.53 | 14.90±0.99 | 19.20±1.93 | 0.73±0.12 | 37.97 | 0.62 | 215 | 75 | 8.77±0.35 | 15.90±0.74 | 24.40±3.17 | 1.09±0.21 | 44.71 |
| 3 | Aliya | KZ | 0.71 | 231 | 113 | 10.00±0.85 | 17.60±1.90 | 32.90±5.51 | 1.32±0.10 | 40.09 | 0.69 | 219 | 105 | 10.42±0.61 | 21±1.15 | 50.3±5.14 | 2.22±0.29 | 44.14 |
| 4 | Azharly | KZ | 0.8 | 225 | 94 | 11.96±0.48 | 20.20±0.63 | 51.60±6.00 | 2.30±0.25 | 44.5 | 0.7 | 218 | 122 | 11.35±0.98 | 19.9±1.45 | 49.6±7.89 | 2.14±0.15 | 43.15 |
| 5 | Akbidaj | KZ | 0.7 | 222 | 89 | 8.14±0.34 | 14.00±0.82 | 28.90±2.60 | 1.06±0.11 | 36.71 | 0.69 | 212 | 112 | 10.1±0.6 | 19.6±1.96 | 40.0±4.19 | 1.82±0.25 | 45.48 |
| 6 | Batyr | KZ | 0.68 | 226 | 63 | 8.51±0.37 | 13.90±0.74 | 37.90±3.48 | 1.61±0.22 | 42.53 | 0.73 | 216 | 133 | 8.92±0.64 | 17±1.05 | 38.40±4.03 | 1.59±0.16 | 41.8 |
| 7 | Egemen 20 | KZ | 0.7 | 228 | 115 | 12.41±0.56 | 19.82±1.40 | 41.45±3.64 | 2.01±0.21 | 48.46 | 0.71 | 225 | 117 | 11.6±0.67 | 17.5±2.07 | 38±13.68 | 1.61±0.74 | 42.37 |
| 8 | Farabi | KZ | 0.69 | 225 | 64 | 8.54±0.36 | 15.50±0.71 | 37.10±6.90 | 1.09±0.26 | 29.43 | 0.6 | 216 | 90 | 9.24±0.87 | 16.7±1.64 | 43.6±7.32 | 1.60±0.32 | 36.7 |
| 9 | Mataj | KZ | 0.61 | 228 | 107 | 12.71±1.36 | 23.00±1.70 | 57.00±13.81 | 2.81±0.65 | 41.97 | 0.77 | 228 | 110 | 9.76±1.09 | 18±1.63 | 36.7±8.83 | 1.28±0.25 | 34.88 |
| 10 | Kyzyl bidaj | KZ | 0.69 | 222 | 70 | 11.99±0.64 | 18.1±0.88 | 44.6±3.72 | 1.62±0.29 | 36.32 | 0.66 | 217 | 119 | 10.37±0.90 | 17.20±1.14 | 35.20±4.94 | 1.57±0.21 | 44.72 |
| 11 | Keremet | KZ | 0.52 | 222 | 68 | 9.55±0.69 | 20.00±2.75 | 33.90±4.91 | 1.47±0.19 | 43.27 | 0.65 | 217 | 98 | 9.81±0.56 | 21.8±1.32 | 55.6±6.77 | 2.42±0.27 | 43.53 |
| 12 | Koksu | KZ | 0.51 | 221 | 75 | 9.95±0.44 | 17.60±0.84 | 42.90±4.72 | 1.99±0.35 | 46.34 | 0.65 | 214 | 103 | 9.92±0.77 | 17.7±1.06 | 47.3±6.65 | 2.49±0.31 | 52.64 |
| 13 | Kokbidaj | KZ | 0.66 | 225 | 77 | 8.00±0.53 | 18.20±0.92 | 41.10±3.03 | 1.89±0.40 | 45.96 | 0.6 | 222 | 85 | 8.76±0.74 | 19.6±1.17 | 56±5.6 | 2.44±0.31 | 43.57 |
| 14 | Karaspan | KZ | 0.71 | 222 | 118 | 10.80±0.59 | 18.30±0.95 | 31.60±4.14 | 1.23±0.15 | 38.86 | 0.7 | 218 | 120 | 10.93±0.72 | 19.5±1.08 | 52.9±7.75 | 2.21±0.42 | 41.78 |
| 15 | Karlygash | KZ | 0.54 | 225 | 98 | 10.55±0.50 | 18.40±1.51 | 36.80±4.37 | 1.44±0.10 | 38.99 | 0.69 | 217 | 103 | 10.94±0.61 | 20.1±1.52 | 55.6±7.95 | 2.16±0.46 | 38.85 |
| 16 | Kazakhstanskaya 10 | KZ | 0.74 | 227 | 119 | 8.70±0.54 | 15.60±0.52 | 24.80±3.39 | 1.13±0.27 | 45.65 | 0.71 | 220 | 122 | 11.67±1.14 | 19±1.63 | 43.5±5.85 | 1.74±0.32 | 40 |
| 17 | Progress | KZ | 0.56 | 220 | 81 | 9.40±0.52 | 14.90±0.88 | 37.60±4.90 | 1.23±0.16 | 32.61 | 0.64 | 218 | 102 | 10.94±1.02 | 18.5±0.85 | 46.9±7.23 | 2.11±0.52 | 44.99 |
| 18 | Prezident | KZ | 0.44 | 226 | 101 | 9.62±0.44 | 18.30±0.67 | 37.10±4.48 | 1.58±0.24 | 42.67 | 0.73 | 224 | 98 | 7.87±0.64 | 17.3±2.06 | 39.4±5.48 | 1.68±0.15 | 42.64 |
| 19 | Raminal | KZ | 0.62 | 226 | 79 | 10.05±0.44 | 18.00±1.05 | 41.70±3.53 | 1.80±0.40 | 43.09 | 0.68 | 220 | 98 | 10.4±0.9 | 19.6±1.51 | 43.6 | 1.85±0.45 | 42.32 |
| 20 | Rasad | KZ | 0.73 | 228 | 80 | 10.35±0.71 | 19.00±1.15 | 48.50±2.72 | 2.24±0.19 | 46.25 | 0.67 | 222 | 101 | 10.39±1.08 | 20.8±1.32 | 51.5±6.17 | 2.21±0.20 | 42.95 |
| 21 | Rausin | KZ | 0.71 | 224 | 92 | 9.55±0.55 | 14.80±0.79 | 38.80±2.90 | 1.46±0.13 | 37.73 | 0.66 | 217 | 105 | 9.45±1.3 | 16.5±1.58 | 37.70±3.09 | 1.39±0.26 | 36.9 |

| | | | | | | | | | | | | | | | | | | |
|---|---|---|---|---|---|---|---|---|---|---|---|---|---|---|---|---|---|---|
| 22 | Reke | KZ | 0.7 | 225 | 85 | 9.15±0.82 | 16.30±1.06 | 37.50±4.93 | 1.37±0.11 | 36.45 | 0.66 | 216 | 104 | 8.64±0.62 | 18±2.16 | 42.1±5.02 | 1.63±0.24 | 38.72 |
| 23 | Zhalyn | KZ | 0.62 | 231 | 94 | 10.08±0.50 | 17.20±1.03 | 46.80±5.03 | 1.70±0.32 | 36.3 | 0.71 | 222 | 103 | 11.64±0.89 | 20.4±1.17 | 51.9±10.65 | 1.64±0.42 | 31.6 |
| 24 | Yuzhnaya 12 | KZ | 0.75 | 230 | 70 | 10.25±0.42 | 18.00±0.94 | 43.70±8.06 | 1.50±0.22 | 34.28 | 0.7 | 222 | 95 | 10.86±0.81 | 19.3±1.34 | 56.1±3.9 | 1.98±0.27 | 35.29 |
| 25 | Pirotriks 50 | KZ | 0.62 | 231 | 103 | 8.27±1.02 | 15.3±3.37 | 33.4±6.54 | 1.14±0.13 | 34.13 | 0.67 | 222 | 110 | 8.89±0.33 | 15.50±0.97 | 39.20±4.24 | 1.56±0.15 | 39.74 |
| 26 | Daulet | KZ | 0.64 | 224 | 115 | 11.66±0.86 | 18.90±1.52 | 38.80±3.08 | 1.39±0.19 | 35.82 | 0.64 | 225 | 113 | 7±0.53 | 13.9±1.1 | 33±5.12 | 0.92±0.3 | 27.88 |
| 27 | Konditerskaya | KZ | 0.58 | 226 | 60 | 9.32±0.95 | 16.1±1.52 | 43.7±9.25 | 1.95±0.39 | 44.62 | 0.64 | 222 | 85 | 9.29±0.96 | 16.80±0.63 | 41.00±2.00 | 1.44±0.05 | 35.15 |
| 28 | 428/MK-122A-1 | KZ | 0.76 | 229 | 96 | 9.20±0.53 | 19.80±0.63 | 46.90±4.58 | 2.00±0.22 | 42.64 | 0.72 | 222 | 105 | 10.05±0.68 | 21.9±0.88 | 46.6±4.58 | 2.13±0.29 | 45.71 |
| 29 | Steklovidnaya 24 | KZ | 0.77 | 223 | 79 | 9.45±1.01 | 16.60±1.71 | 42.00±6.45 | 1.68±0.32 | 39.9 | 0.68 | 213 | 88 | 9.18±0.65 | 18.6±1.9 | 53.5±6.36 | 2.27±0.28 | 42.43 |
| 30 | Sultan 2 | KZ | 0.83 | 228 | 115 | 13.88±2.5 | 24.5±3.1 | 70.8±13.88 | 3.01±0.83 | 42.51 | 0.75 | 220 | 111 | 13.75±0.95 | 20.90±2.18 | 50.90±6.57 | 1.68±0.26 | 32.99 |
| 31 | Naz/Immun 78 | KZ | 0.74 | 223 | 87 | 7.62±0.64 | 19.8±1.75 | 47.7±7.27 | 1.85±0.38 | 38.78 | 0.73 | 217 | 93 | 6.68±0.40 | 15.50±0.97 | 39.90±4.04 | 1.52±0.12 | 41.3 |
| 32 | Naz/GF 55-2 | KZ | 0.78 | 228 | 125 | 10.66±0.43 | 18.20±0.42 | 40.10±3.31 | 1.50±0.16 | 37.31 | 0.69 | 222 | 128 | 12.11±1.16 | 21.7±1.16 | 46.3±6.82 | 1.68±0.35 | 36.29 |
| 33 | Naz/GF 55-3 | KZ | 0.74 | 224 | 86 | 9.78±0.73 | 19.1±1.73 | 41.2±7.05 | 1.62±0.25 | 39.32 | 0.69 | 221 | 90 | 9.16±0.47 | 16.40±0.97 | 34.70±12.24 | 1.41±0.28 | 40.75 |
| 34 | Naz/GF 55-5 | KZ | 0.81 | 225 | 106 | 10.7±0.86 | 19.9±1.2 | 39.5±3.63 | 1.4±0.27 | 35.44 | 0.73 | 222 | 98 | 9.20±0.74 | 14.80±0.79 | 38.30±5.29 | 1.47±0.30 | 38.46 |
| 35 | Yr/Octyabrina | KZ | 0.8 | 227 | 112 | 11.37±1.1 | 20.1±2.02 | 43.1±9.19 | 1.72±0.48 | 39.91 | 0.73 | 222 | 115 | 9.49±0.59 | 16.80±1.23 | 37.10±4.89 | 1.52±0.13 | 40.92 |
| 36 | 425/Obri | KZ | 0.86 | 230 | 119 | 11.35±0.61 | 17.90±0.88 | 41.80±4.85 | 1.55±0.17 | 37.02 | 0.78 | 225 | 125 | 12±1.82 | 20.5±1.72 | 47.9±8.88 | 1.74±0.45 | 36.33 |
| 37 | Alihan | KZ | 0.67 | 221 | 79 | 8.00±0.58 | 16.10±1.10 | 38.10±4.86 | 1.78±0.18 | 46.77 | 0.69 | 212 | 102 | 8.45±0.95 | 17.6±2.41 | 44.2±6.36 | 2.1±0.5 | 47.51 |
| 38 | Anar | KZ | 0.75 | 230 | 86 | 9.50±0.47 | 17.00±0.82 | 37.10±4.33 | 1.48±0.18 | 39.76 | 0.72 | 219 | 77 | 11.61±0.9 | 22.2±1.55 | 49.9±5.95 | 1.96±0.33 | 39.28 |
| 39 | Derbes | KZ | 0.62 | 221 | 84 | 8.25±0.42 | 17.50±1.43 | 30.40±5.17 | 1.00±0.26 | 32.73 | 0.68 | 215 | 107 | 8.29±0.45 | 17.30±1.06 | 36.5±3.87 | 1.29±0.29 | 35.21 |
| 40 | Krasnovodapadskaya 210 | KZ | 0.61 | 225 | 74 | 9.35±0.72 | 19.10±1.52 | 46.90±6.40 | 1.69±0.46 | 35.97 | 0.73 | 226 | 85 | 8.62±0.68 | 16.6±1.58 | 33.8±7.74 | 1.23±0.14 | 36.39 |
| 41 | APK/Progress | KZ | 0.79 | 225 | 102 | 10.07±0.43 | 18.70±0.67 | 42.50±3.78 | 1.75±0.11 | 41.13 | 0.73 | 222 | 108 | 10.26±0.93 | 21.3±1.89 | 45.7±5.64 | 1.86±0.14 | 40.77 |
| 42 | Almaly/Orbij | KZ | 0.76 | 225 | 91 | 8.29±0.52 | 19.5±2.07 | 42.3±5.17 | 1.94±0.27 | 45.86 | 0.75 | 221 | 94 | 8.27±0.85 | 17.20±1.23 | 39.30±5.79 | 1.54±0.25 | 39.29 |
| 43 | Gozgon | IWWIP | 0.62 | 224 | 86 | 8.93±0.62 | 16.60±1.07 | 42.90±5.65 | 2.05±0.19 | 47.69 | 0.75 | 223 | 91 | 9.46±0.59 | 18.7±1.64 | 45.5±7.88 | 2.15±0.33 | 47.25 |
| 44 | Bunyodkor | IWWIP | 0.64 | 223 | 85 | 9.18±0.30 | 18.50±0.53 | 36.70±6.15 | 1.49±0.22 | 40.71 | 0.8 | 215 | 90 | 9.89±1.16 | 20.5±2.01 | 45.2±10.63 | 1.72±0.58 | 38.05 |
| 45 | Faravon | IWWIP | 0.69 | 227 | 92 | 9.18±0.62 | 17.70±0.67 | 50.20±6.68 | 2.12±0.13 | 42.25 | 0.75 | 221 | 93 | 9.2±0.79 | 19.2±1.93 | 51.9±9.17 | 2.09±0.14 | 40.33 |
| 46 | Hazrati Bashir | IWWIP | 0.61 | 221 | 73 | 10.48±0.56 | 17.30±0.67 | 50.00±8.04 | 1.57±0.17 | 31.32 | 0.7 | 214 | 77 | 10.48±0.56 | 17.3±0.67 | 50±8.04 | 1.57±0.17 | 31.4 |
| 47 | Hisorok | IWWIP | 0.63 | 227 | 69 | 8.96±0.64 | 19.60±1.35 | 48.40±6.11 | 2.09±0.11 | 43.24 | 0.7 | 220 | 73 | 9.49±1.06 | 20.5±2.32 | 51.1±9.96 | 2.15±0.38 | 42.07 |

| | | | | | | | | | | | | | | | | | | |
|---|---|---|---|---|---|---|---|---|---|---|---|---|---|---|---|---|---|---|
| 48 | Layagatlii 80 | IWWIP | 0.64 | 225 | 109 | 11.62±0.37 | 22.2±1.03 | 62.8±5.96 | 2.54±0.24 | 40.45 | 0.73 | 216 | 103 | 10.13±0.86 | 19.50±1.08 | 49.60±8.50 | 1.61±0.34 | 32.54 |
| 49 | Shafag 2 | IWWIP | 0.62 | 225 | 103 | 11.95±0.6 | 21±0.94 | 58.6±3.75 | 2.47±0.29 | 42.15 | 0.69 | 225 | 97 | 9.46±0.67 | 18.10±1.10 | 43.20±4.73 | 1.65±0.22 | 38.17 |
| 50 | Egana | IWWIP | 0.64 | 224 | 80 | 8.07±0.46 | 17.30±1.25 | 44.20±7.04 | 1.86±0.19 | 41.97 | 0.7 | 224 | 85 | 8.67±0.45 | 17.20±1.14 | 55.1±6.61 | 2.34±0.32 | 42.47 |
| 51 | 415-SP-2 | KZ/C | 0.71 | 224 | 88 | 10.12±0.75 | 19.40±0.89 | 46.1±3.31 | 1.75±0.26 | 37.96 | 0.62 | 220 | 77 | 11.73±1.13 | 19.71±1.70 | 47.90±4.23 | 2.08±0.24 | 43.32 |
| 52 | 416-SP-2 | KZ/C | 0.71 | 224 | 88 | 9.70±1.59 | 15.57±2.70 | 42.30±3.77 | 1.55±0.21 | 36.74 | 0.57 | 218 | 74 | 10.65±1.02 | 16.29±2.43 | 44.30±6.20 | 1.98±0.22 | 44.65 |
| 53 | 2-CP | KZ/C | 0.73 | 223 | 85 | 8.86±1.02 | 17.60±1.26 | 43.60±10.17 | 1.63±0.46 | 37.45 | 0.73 | 212 | 94 | 10.39±0.62 | 20.60±1.78 | 56.30±5.54 | 1.80±0.31 | 31.90 |
| 54 | 3-CP | KZ/C | 0.76 | 227 | 115 | 9.55±0.78 | 18.70±1.25 | 33.50±5.93 | 1.30±0.32 | 38.81 | 0.77 | 224 | 137 | 11.60±1.02 | 20.60±1.17 | 52.60±6.02 | 2.52±0.58 | 47.95 |
| 55 | 4-CP | KZ/C | 0.7 | 218 | 96 | 8.51±0.94 | 14.40±1.51 | 33.89±6.85 | 1.31±0.26 | 38.60 | 0.69 | 210 | 120 | 9.64±0.61 | 18.10±0.99 | 43.10±5.74 | 2.06±0.19 | 47.68 |
| 56 | 5-CP | KZ/C | 0.73 | 224 | 98 | 9.97±0.31 | 18.10±0.74 | 42.60±4.62 | 1.91±0.31 | 44.93 | 0.76 | 220 | 115 | 10.92±0.49 | 20.60±0.70 | 48.70±4.06 | 2.14±0.20 | 43.94 |
| 57 | 6-CP | KZ/C | 0.71 | 224 | 97 | 9.64±0.93 | 15.80±1.14 | 36.90±5.61 | 1.51±0.21 | 41.00 | 0.74 | 218 | 125 | 11.30±0.80 | 18.80±1.03 | 46.00±6.18 | 2.21±0.32 | 47.93 |
| 58 | 7-CP | KZ/C | 0.7 | 228 | 94 | 11.16±0.66 | 18.80±1.23 | 44.80±3.77 | 1.95±0.21 | 43.42 | 0.74 | 221 | 127 | 12.13±0.77 | 20.80±1.14 | 48.10±3.41 | 2.39±0.22 | 49.65 |
| 59 | 8-CP | KZ/C | 0.74 | 227 | 86 | 8.79±0.63 | 18.90±1.45 | 33.70±4.06 | 1.08±0.26 | 32.08 | 0.73 | 217 | 107 | 8.96±0.26 | 19.40±1.17 | 39.50±5.28 | 1.39±0.18 | 35.16 |
| 60 | 9-CP | KZ/C | 0.47 | 226 | 87 | 9.16±0.70 | 19.40±1.43 | 45.50±4.45 | 2.07±0.27 | 45.49 | 0.72 | 217 | 137 | 8.89±0.44 | 20.50±.43 | 44.60±3.47 | 2.01±0.39 | 45.09 |
| 61 | 10-CP | KZ/C | 0.77 | 226 | 120 | 10.11±0.86 | 20.20±2.10 | 46.60±3.60 | 1.93±0.13 | 41.42 | 0.79 | 218 | 110 | 9.66±0.57 | 20.20±2.49 | 38.80±9.45 | 1.68±0.29 | 43.27 |
| 62 | 11-CP | KZ/C | 0.74 | 228 | 87 | 10.22±0.70 | 19.44±1.33 | 46.78±8.27 | 1.93±0.53 | 41.26 | 0.71 | 218 | 102 | 10.23±0.55 | 20.80±1.23 | 48.10±4.63 | 1.79±0.32 | 37.19 |
| 63 | 12-CP | KZ/C | 0.75 | 225 | 97 | 10.30±0.72 | 20.50±1.58 | 42.80±5.07 | 1.52±0.26 | 35.58 | 0.7 | 221 | 135 | 12.24±0.63 | 23.90±1.32 | 64.50±10.90 | 2.64±0.61 | 40.98 |
| 64 | 13-CP | KZ/C | 0.71 | 219 | 94 | 7.54±1.27 | 17.20±2.04 | 44.30±5.76 | 1.82±0.25 | 41.02 | 0.75 | 217 | 129 | 9.73±1.84 | 20.70±1.06 | 49.80±6.34 | 2.00±0.29 | 40.16 |
| 65 | 14-CP | KZ/C | 0.7 | 222 | 115 | 10.07±0.97 | 18.90±1.37 | 46.10±7.81 | 1.65±0.31 | 35.86 | 0.66 | 209 | 110 | 12.50±0.94 | 21.50±2.01 | 54.50±5.34 | 2.24±0.30 | 41.17 |
| 66 | 15-CP | KZ/C | 0.75 | 222 | 101 | 10.90±0.97 | 22.20±1.32 | 37.90±2.64 | 1.46±0.12 | 38.47 | 0.75 | 219 | 128 | 11.45±0.66 | 21.50±1.51 | 52.90±3.14 | 1.96±0.42 | 36.98 |
| 67 | 16-CP | KZ/C | 0.73 | 224 | 108 | 10.15±0.56 | 19.00±1.94 | 36.90±2.56 | 1.38±0.15 | 37.32 | 0.7 | 218 | 110 | 9.69±0.65 | 19.30±0.95 | 42.60±2.99 | 1.67±0.12 | 39.10 |
| 68 | 17-CP | KZ/C | 0.67 | 221 | 97 | 11.95±0.90 | 20.30±1.49 | 41.20±6.46 | 1.59±0.50 | 38.52 | 0.76 | 222 | 125 | 11.92±0.55 | 20.80±1.03 | 44.30±4.42 | 1.58±0.27 | 35.69 |
| 69 | 18-CP | KZ/C | 0.73 | 218 | 115 | 10.09±0.45 | 21.00±0.67 | 34.80±3.94 | 1.10±0.36 | 31.58 | 0.75 | 218 | 131 | 9.63±0.23 | 21.10±0.57 | 43.60±4.67 | 1.43±0.12 | 32.72 |
| 70 | 19-CP | KZ/C | 0.61 | 226 | 94 | 9.52±1.21 | 19.40±1.96 | 33.90±6.95 | 1.15±0.30 | 33.89 | 0.77 | 225 | 85 | 10.56±1.25 | 19.40±2.22 | 45.50±7.21 | 1.32±0.22 | 29.08 |

Note: KZ – Kazakhstan; IWWIP (International Winter Wheat Improvement Program) developed by CIMMYT-ICARDA; KZ/C – Kazakhstan-CIMMYT breeding program; NDVI - normalized difference vegetation index; PH, cm - plant height, DH - days to heading, SL, cm - spike lengths, SS - the mean number of spikelets/spike, GS - grains/spike, WGS, g - the weight of grain/spike, TKW, g - thousand kernel weights

**Table S3**. Pedigree of promising winter wheat lines, originating from Kazakhstan-CIMMYT breeding program.

| # | Name | Pedigree |
|---|---|---|
| 51 | 415-SP-2 | 1454/309/1018(104(N91/5347)/ Mereke))//Mereke/1101-17/309 |
| 52 | 416-SP-2 | 1454/309/1018(104(N91/5347)/Mereke))//Mereke/1101-17/309 |
| 53 | 2-CP | 1300(93.AUS 4930.7/2*PASTOR/4/338-K1-1// ANB/BUC/3/GS50A/5/TAM200/KAUZ/Naz/367/113/DO-4 DS/366 |
| 54 | 3-CP | 23/Kupava//1774/23 |
| 55 | 4-CP | 23/Kupava//1774/23 |
| 56 | 5-CP | 20/Umanka//1773/22 |
| 57 | 6-CP | 23/Kupava//1773/22 |
| 58 | 7-CP | 179/ 1760 |
| 59 | 8-CP | 179/586(Bermet)// RWKLDN9 |
| 60 | 9-CP | Ramin/1736/1594 (1013(97(N20 /Umanka)/ Egemen)//Brundage 96/23 Brundage 96 |
| 61 | 10-CP | 1777Darya/1724 /1581/ (807 (Naz/Umanka)/Almaly)//Zimorodok/Almaly |
| 62 | 11-CP | 1777Darya/1737/1014 (99(Madsen/Cm.24)/Arap)//Brundage 96/23Brundage 96 |
| 63 | 12-CP | 1772Viza/Almaly//Viza |
| 64 | 13-CP | 23/Kupava//Ulugbek/Yr4/Mereke/197 |
| 65 | 14-CP | 1320 (113.338-K1-1//ANB/BUC/3/GS50A/4 /TREGO/JGR 8W/5/TX69A509-2//BBY2/ FOX/3/PKL70/LIRA/4/YMH/TOB// MCD/3/LIRA)/Tungysh)//323 |
| 66 | 15-CP | 1214. MV10-2000/4/AGRI/NAC// KAUZ/3/ 1D13.1/MLT//Tungysh/Egemen |
| 67 | 16-CP | 11Fielder/269//88 |
| 68 | 17-CP | 1046 DALNITSKAYA/4/AGRI/NAC//KAUZ/3/1D13.1/MLT/5/F10S-1//ATAY/GAL-VEZ87//Amigo |
| 69 | 18-CP | 845/ Kupava/1654/1022 (108 (Vabah 1( 5351)/133)/Arap)/Arap)//Arap |
| 70 | 19-CP | 1286(79 (ARDEAL/BOEMA//F135U2-1/5/ TX69A509-2//BBY2/FOX/3/PKL70 /LIRA/4/YMH/TOB//MCD/3/LIRA)/Naz)/Naz//296 W2691SrTt-1 Cl 17385 |

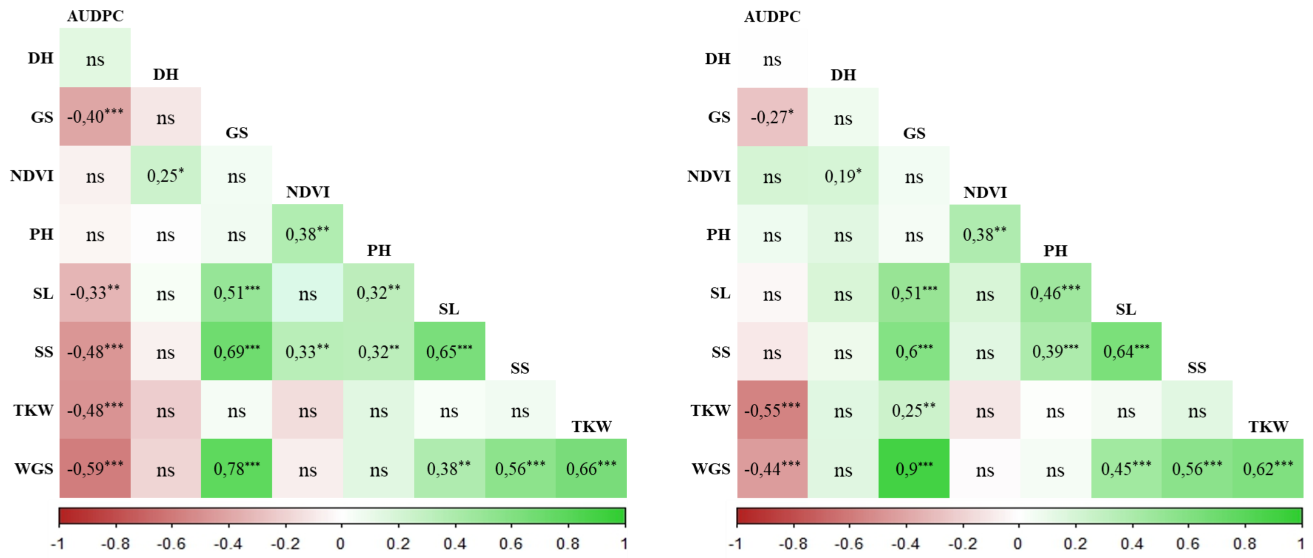


(**a**) (**b**)

**Figure S1**. Pearson correlation analysis between the disease progression value (AUDPC) and the main indicators of wheat productivity in 2021 (a) and 2022 (b).
Note: *** – p<0.001; ** – p<0.01; * – p<0.05; ns – not significant.

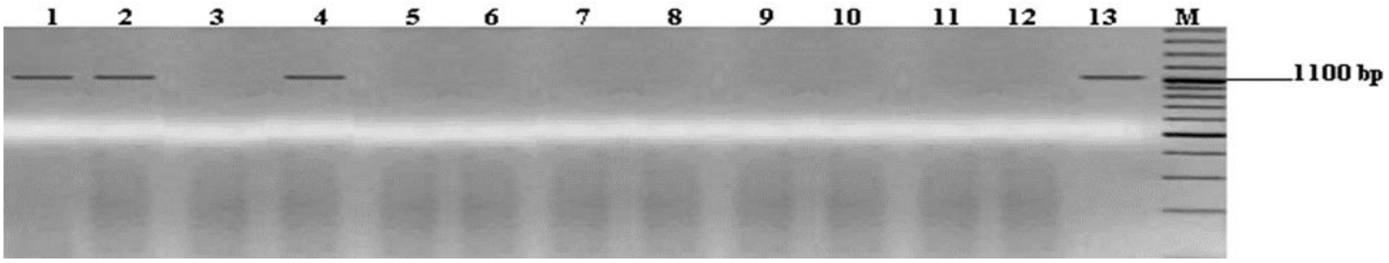


1-Anar, 2-Mataj, 3-Alatau, 4-Raminal, 5-Almaly, 6-Aliya, 7-Akbidaj, 8-Batyr, 9-Egemen 20, 10-Farabi, 11-Kyzyl bidaj, 12-ddH2O (negative control), 13- Transfer/6*TC (positive control), M - molecular weight marker (Gene Ruler, 100 bp DNA Ladder)

**Figure S2.** DNA amplification products of wheat entries using primers to the STS J13 locus linked with the *Lr9* resistance gene. The sizes of the bands for *Lr9* are 1100 bp. The arrows show the band size of *Lr9*-carrying germplasm (1, 2, 4 and 13 – positive control).

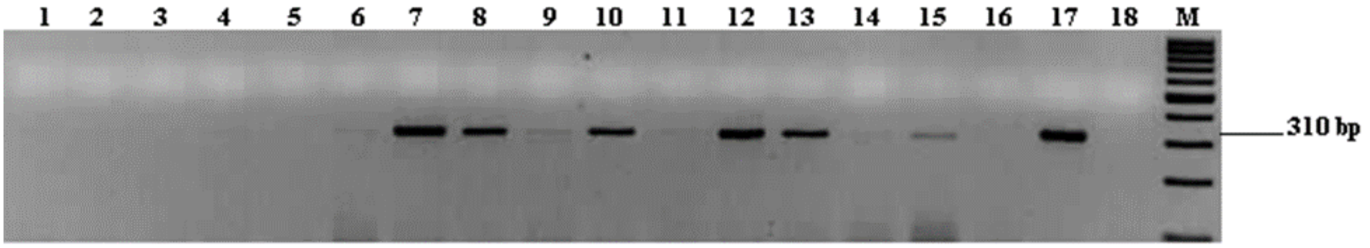


1-Alatau, 2-Almaly, 3-Azharly, 4-Akbidaj, 5-Batyr, 6-Egemen 20, 7-Aliya, 8-Hazrati Bashir, 9-Farabi, 10-Layagatlii 80, 11-Mataj, 12-Egana, 13-Hisorok, 14-Koksu, 15-Yr/Octyabrina, 16-Kyzyl Bidaj, 17- TC*6/Exchange (positive control), 18- ddH2O (negative control), M - molecular weight marker (Gene Ruler, 100 bp DNA Ladder)

**Figure S3.** DNA amplification products of wheat entries using primers to the STS *Lr 10* locus linked with the *Lr10* resistance gene. The sizes of the bands for *Lr10* are 310 bp. The arrows show the band size of *Lr10*-carrying germplasm (7, 8, 10, 12, 13, 15 and 17-positive control).

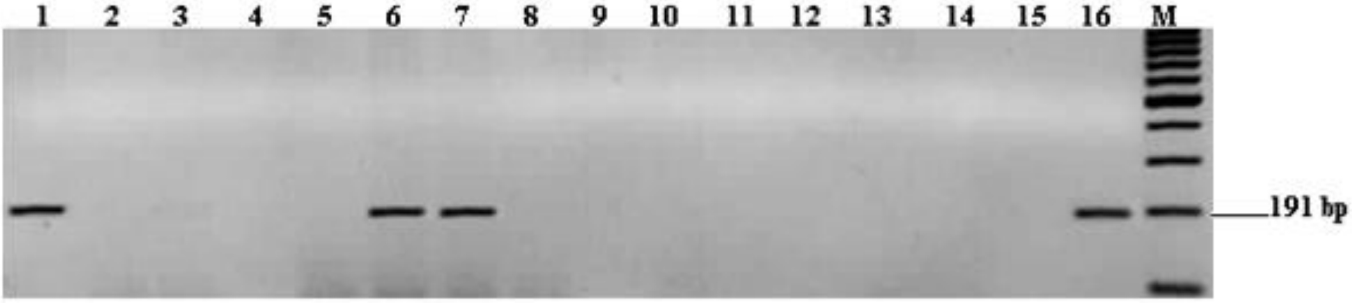


1-Kyzyl bidaj, 2-Zhalyn, 3-Koksu, 4-Kokbidaj, 5-Karaspan, 6-Keremet, 7-Hisorok, 8-Karlygash, 9-Kazakhstanskaya 10, 10-Progress, 11-Prezident, 12-Raminal, 13-Rasad, 14-Rausin, 15- ddH2O (negative control), 16- TC*7/Tr (positive control), M - molecular weight marker (Gene Ruler, 100 bp DNA Ladder)

**Figure S4.** DNA amplification products of wheat entries using primers to the STS PSY_EF2/PSY_ER4 locus linked with the *Lr19* resistance gene. The sizes of the bands for *Lr19* are 191 bp. The arrows show the band size of *Lr19*-carrying germplasm (1,6,7 and 16-positive control).

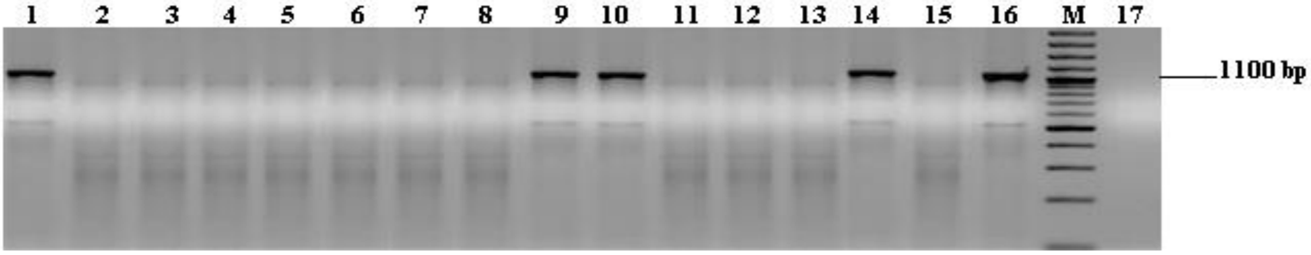


1-Keremet, 2-Koksu, 3-Kokbidaj, 4-Karaspan, 5-Karlygash, 6-Kazakhstanskaya 10, 7-Progress, 8-Prezident, 9-Bunyogkor, 10-Layagatlii 80, 11-Raminal, 12-Rasad, 13-Rausin, 14-Shafag 2, 15-Reke, 16- TC*6/ST-1-25 (positive control), M- molecular weight marker (Gene Ruler, 100 bp DNA Ladder), 17- ddH2O (negative control)

**Figure S5**. DNA amplification products of wheat entries using primers to the STS Iag 95 locus linked with the *Lr26* resistance gene. The sizes of the bands for *Lr26* are 1100 bp. The arrows show the band size of *Lr26*-carrying germplasm (1, 9, 10, 14 and 16-positive control).

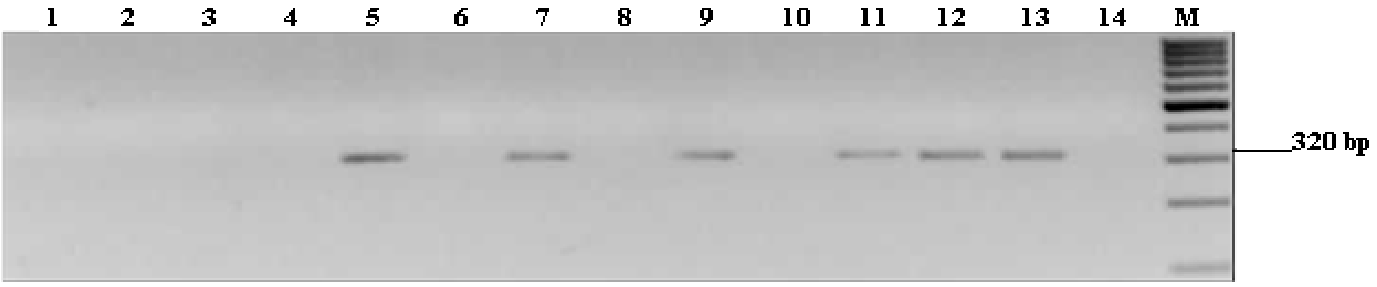


1-Batyr, 2-Zhalyn, 3-Daulet, 4-Yuzhnaya 12, 5-Aliya, 6-Gozgon, 7-Pirotriks 50, 8-Hazrati Bashir, 9-Reke, 10-Egana, 11-Konditerskaya, 12-425/Obri, 13- CS2D-2M (positive control), 14-ddH2O (negative control), M- molecular weight marker (Gene Ruler, 100 bp DNA Ladder)

**Figure S6.** DNA amplification products of wheat entries using primers to the SSR wmc313 locus linked with the *Lr28* resistance gene. The sizes of the bands for *Lr28* are 320 bp. The arrows show the band size of *Lr28*-carrying germplasm (5, 7, 9, 11, 12 and 13-positive control).

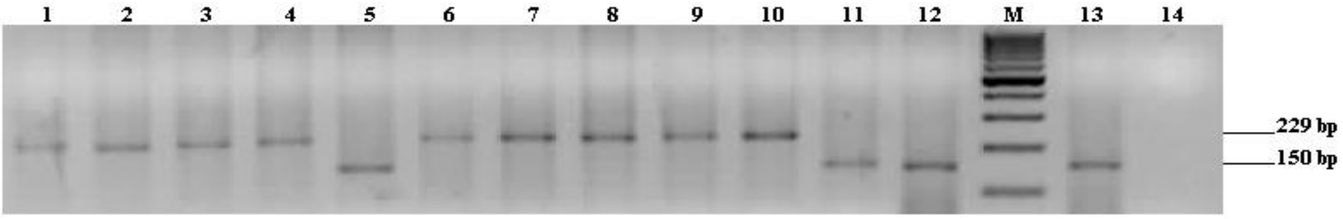


1- Naz/GF-55-2, 2-Naz/GF-55-3, 3-Yr/Octyabrina, 4-Alihan, 5-Almaly, 6-425/Obri, 7-Anar, 8-Derbes, 9-Krasnovodopadskaya 210, 10-Almatinskaya polukarlikovaya/Progress, 11-Karaspan, 12-Karlygash, M- molecular weight marker (Gene Ruler, 100 bp DNA Ladder), 13- TC*6/PI58548 (positive control), 14- ddH2O (negative control)

**Figure S7.** DNA amplification products of wheat entries using primers to the STS csLV34 locus linked with the *Lr34* resistance gene. The arrows show the band size of *Lr34*-carrying and *Lr34*-none-carrying. The sizes of the bands are 150 bp for *Lr34* (5, 11, 12 and 13-positive control) and 229 bp for non-carryes of *Lr34* (1, 2, 3, 4, 6, 7, 8, 9, 10)

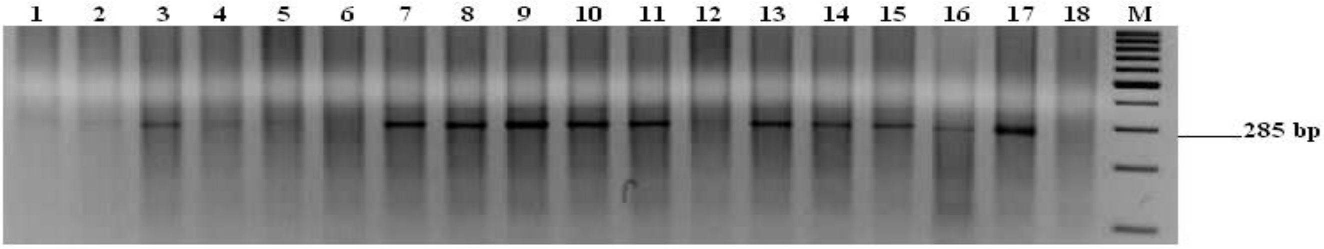


1- Shafag 2, 2-Egana, 3-Aliya, 4-Egemen 20, 5-Steklovidnaya 24, 6-Sultan 2, 7-Mataj, 8-Kyzyl bidaj, 9-Keremet, 10-Kokbidaj, 11-Rasad, 12-Naz/Immun 78, 13-Reke, 14-Hisorok, 15-Alihan, 16-Krasnovodopadskaya 210, 17- TC*6/VPM (positive control), 18-ddH2O (negative control), M - molecular weight marker (Gene Ruler, 100 bp DNA Ladder)

**Figure S8.** DNA amplification products of wheat entries using primers to the CAPS URIC-LN2 linked with the *Lr37* resistance gene. The sizes of the bands for *Lr37* are 285 bp. The arrows show the band size of *Lr37*-carrying germplasm (3, 7, 8, 9, 10, 11, 13, 14, 15, 16 and 17-positive control).

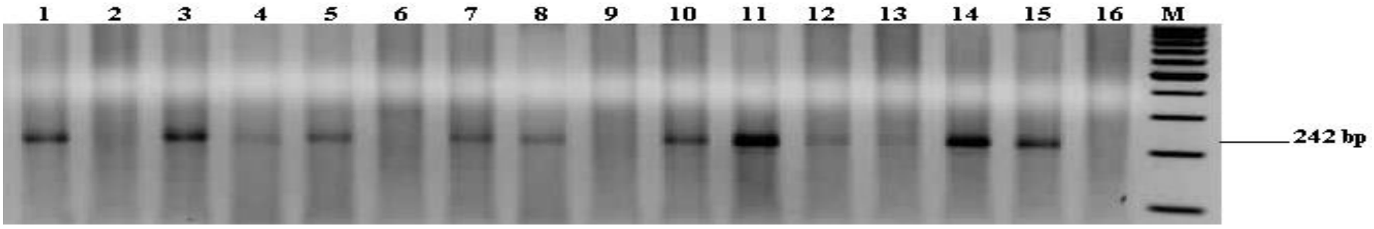


1-Alatau, 2-Alihan, 3-Azharly, 4-Anar, 5-Akbidaj, 6-Derbes, 7-Batyr, 8-Farabi, 9-Krasnovodopadskaya 210, 10-Mataj, 11-Keremet, 12-Almatinskaya polukarlikovaya/Progress, 13-Almaly/Obri, 14-Koksu, 15- Pavon 76 (positive control), 16- ddH2O (negative control), M - molecular weight marker (Gene Ruler, 100 bp DNA Ladder)

**Figure S9.** DNA amplification products of wheat entries using primers to the STS Xwmc 44 locus linked with the *Lr46* resistance gene. The sizes of the bands for *Lr46* are 242 bp. The arrows show the band size of *Lr46*-carrying germplasm (1, 3, 5, 7, 8, 10, 11, 14 and 15-positive control).

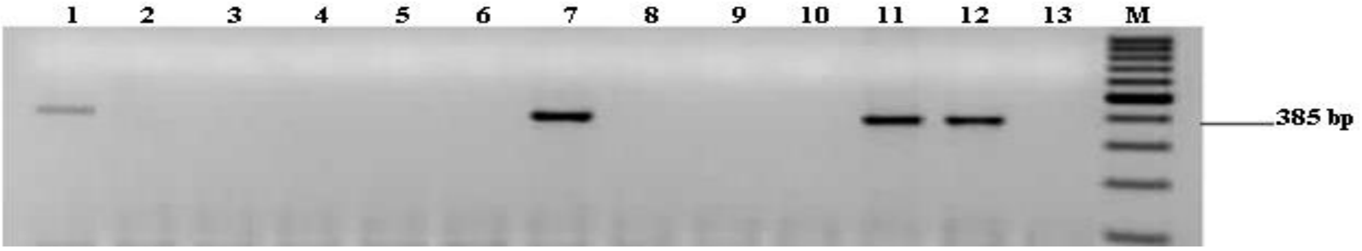


1-Almaly/Obri, 2-Azharly, 3-Koksu, 4-Kokbidaj, 5-Karaspan, 6-Zhalyn, 7-Naz/GF-55-3, 8-Karlygash, 9-Kazakhstanskaya 10, 10-Progress, 11-Alihan, 12- Parula (positive control), 13-ddH2O (negative control), M - molecular weight marker (Gene Ruler, 100 bp DNA Ladder)

**Figure S10.** DNA amplification products of wheat entries using primers to the STS csGS F1 csGS R-1 locus linked with the *Lr68* resistance gene. The sizes of the bands for *Lr68* are 385 bp. The arrows show the band size of *Lr68*-carrying germplasm (1, 7, 11 and 12-positive control)
